# Genome analysis of *Diploscapter coronatus*: insights into molecular peculiarities of a nematode with parthenogenetic reproduction

**DOI:** 10.1186/s12864-017-3860-x

**Published:** 2017-06-24

**Authors:** Hideaki Hiraki, Hiroshi Kagoshima, Christopher Kraus, Philipp H. Schiffer, Yumiko Ueta, Michael Kroiher, Einhard Schierenberg, Yuji Kohara

**Affiliations:** 1 Genome Biology Laboratory, National Institute of Genetics, Mishima, Japan; 2 Zoologisches Institut, Universität zu Köln, Cologne, NRW Germany; 3 Transdisciplinary Research Integration Center, Research Organization of Information and Systems, Tokyo, Japan

**Keywords:** Genome assembly, Allelic expression, Nematode, Parthenogenesis, Meiosis, Cohesin

## Abstract

**Background:**

Sexual reproduction involving the fusion of egg and sperm is prevailing among eukaryotes. In contrast, the nematode *Diploscapter coronatus*, a close relative of the model *Caenorhabditis elegans*, reproduces parthenogenetically. Neither males nor sperm have been observed and some steps of meiosis are apparently skipped in this species. To uncover the genomic changes associated with the evolution of parthenogenesis in this nematode, we carried out a genome analysis.

**Results:**

We obtained a 170 Mbp draft genome in only 511 scaffolds with a N_50_ length of 1 Mbp. Nearly 90% of these scaffolds constitute homologous pairs with a 5.7% heterozygosity on average and inversions and translocations, meaning that the 170 Mbp sequences correspond to the diploid genome. Fluorescent staining shows that the *D. coronatus* genome consists of two chromosomes (2n = 2). In our genome annotation, we found orthologs of 59% of the *C. elegans* genes. However, a number of genes were missing or very divergent. These include genes involved in sex determination (e.g. *xol-1*, *tra-*2) and meiosis (e.g. the kleisins *rec-8* and *coh-3/4*) giving a possible explanation for the absence of males and the second meiotic division. The high degree of heterozygosity allowed us to analyze the expression level of individual alleles. Most of the homologous pairs show very similar expression levels but others exhibit a 2–5-fold difference.

**Conclusions:**

Our high-quality draft genome of *D. coronatus* reveals the peculiarities of the genome of parthenogenesis and provides some clues to the genetic basis for parthenogenetic reproduction. This draft genome should be the basis to elucidate fundamental questions related to parthenogenesis such as its origin and mechanisms through comparative analyses with other nematodes. Furthermore, being the closest outgroup to the genus *Caenorhabditis*, the draft genome will help to disclose many idiosyncrasies of the model *C. elegans* and its congeners in future studies.

**Electronic supplementary material:**

The online version of this article (doi:10.1186/s12864-017-3860-x) contains supplementary material, which is available to authorized users.

## Background

Sexual reproduction including meiosis and subsequent mating is a characteristic feature of eukaryotes allowing them to maximize the diversity of their gene pools and preserve genetic variability. However, sexual reproduction is costly, because recombination breaks up favorable gene combinations faster than it creates new ones [1], and males require significant resources to be produced but do not generate offspring themselves. Although selected representatives of different animal phyla can reproduce parthenogenetically, in most cases this is a facultative feature, depending on environmental conditions. If these are favorable, only parthenogenetic females are found, while under stress they switch to bisexual reproduction [2]. Parthenogenesis has been considered an evolutionary dead end, since it results in the accumulation of deleterious mutations in an irreversible manner known as “Muller’s ratchet” [3]. Furthermore, parthenogenetic reproduction should impair adaptability to environmental change because positive mutations present in different individuals will rarely come together within the same individual, eventually leading to extinction [4, 5]. However, certain cases in nature are in conflict with such a scenario. For example, bdelloid rotifers are thought to have followed parthenogenetic reproduction for millions of years without males and meiosis. Similar cases have been reported for certain ostracods, mites and root-knot nematodes [6]. The bdelloid rotifer genome was sequenced recently and it has been claimed that the homogenizing and diversifying roles of sex may have been compensated there by gene conversion and horizontal gene transfer [7, 8]. However, there are reports on the possibility of infrequent sexual reproduction and atypical meiosis in some bdelloid rotifers [9–11]. The plant parasitic root-knot nematode *Meloidogyne incognita* is a mitotic parthenogen, yet this species is an extremely potent and widely distributed plant parasite. Genome sequencing of *M. incognita* and several related nematodes has been accomplished [12–14]. A series of unusual features were observed in the genome, which are probably linked to plant-parasitic lifestyle, absence of meiosis and parthenogenetic reproduction [15].

Among nematodes, various modes of reproduction are found: dioecy (male and female), androdioecious hermaphrodites (self-fertilizing female) as well as parthenogenesis (development from unfertilized eggs). One such parthenogen is the Rhabditid species *Diploscapter coronatus*, a close outgroup to the model genus *Caenorhabditis*. Many parthenogenetic taxa show infrequent occurrences of males, but in *D. coronatus* no males have been reported under laboratory conditions [16], even under thermal stress increasing the occurrence of males in *C. elegans* and other nematodes [17, 18]. A distinct feature of *D. coronatus* is its truncated meiosis: In contrast to *C. elegans* (and other dioecious or hermaphroditic nematodes) with its two consecutive meiotic divisions, polar body extrusion takes place only once [16]. Thus, *D. coronatus* appears to skip some steps in meiosis. As no parthenogenetic species has been found in the genus *Caenorhabditis* and as *Diploscapter* belongs to the closest outgroup, it is an attractive target to study the genetic basis of parthenogenesis. Moreover, *D. coronatus* is phylogenetically located between *C. elegans* and the well-studied satellite system, *Pristionchus pacificus* [19, 20]. A genomic comparison between *D. coronatus* and the two nematode model organisms will allow a deeper understanding of evolutionary changes on the cellular and molecular level.

## Results

### Genome assembly revealed the paired structure of the *D. coronatus* genome

Initially, we obtained more than 270,000 ESTs (expressed sequence tags, or end sequences of cDNA clones). These were classified into about 13,000 groups based on the 3′ end sequence comparison (see Methods), 48% of which showed strong homology with *C. elegans* proteins, and interestingly most of the ESTs showed clear heterozygosity within the groups. We assume that this heterozygosity is associated with parthenogenetic reproduction, where allelic differences are accumulated and maintained like in somatic cell lines [21], or originated from interspecies hybridization [5]. Subsequently, we obtained genome shotgun reads by various methods from Sanger to next generation sequencing together with fosmid end sequences (Additional file 1: Table S1). All sequence reads were assembled with the Celera assembler [22] resulting in a total genome span of 170 Mbp (511 scaffolds, N_50_: 1.0Mbp) (Table 1). This value is consistent with the genome size calculated from the k-mer distribution (Additional file 2).

**Table 1:** Statistics of the genome assembly

	Number	Length (bp)		
		Total	Max.	N_50_
Scaffolds	511	170,470,384	3,561,896	1,007,652
Contigs	867	169,424,175	1,740,259	487,148
Paired regions^a^	6690	152,151,424	250,158	
SNVs^b^	8,685,973			
In/Dels^b^	997,343			

^a^Homologous regions identified by all-vs.-all scaffold alignment. See Methods for details

^b^Numbers detected in the 152 Mbp paired regions

**Fig. 1:**
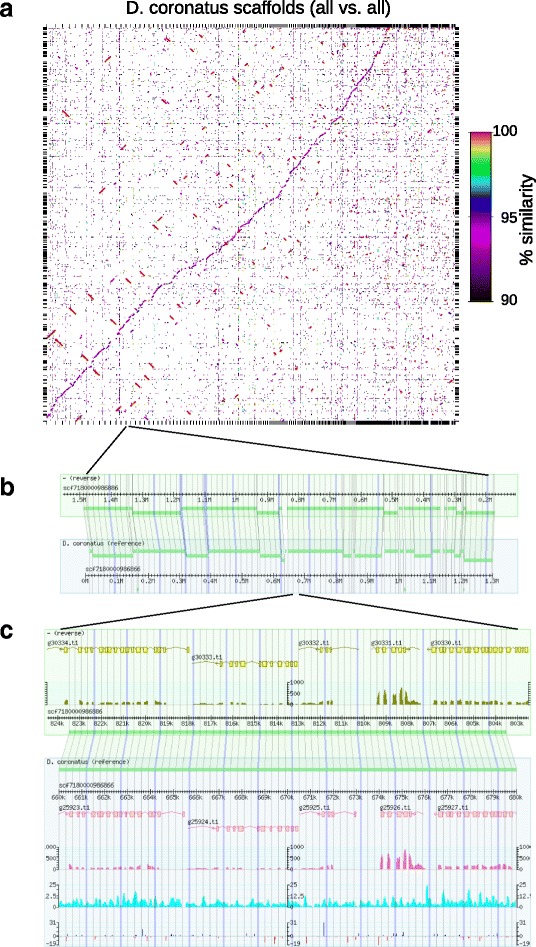
Paired structure of the *D. coronatus* genome. **a** Dot plot of all-vs.-all comparison of the scaffold sequences. Alignments with 90% or more identity are plotted. For most sequences, *purple line* fragments are found indicating the presence of partner sequences with about 94% identity. Both axes are ordered to emphasize paired structure of the genome by MUMmer. Along the X-axis the scaffolds that have homologous counterparts are ordered by length (longest first), and along the Y-axis the corresponding counterpart scaffolds are arranged. *Red lines* indicate trivial hits to themselves. **b** A long syntenic region (1.3Mbp) is visualized using the GBrowse syntenic browser. 65% of the scaffold scf7180000986866 (*lower box*) and its homologous region in the scaffold scf7180000986886 (*upper box*) are shown. The *thick green bars* indicate sequences that have homologous counterparts; these are linked by *light green* shading. A few unpaired short bars indicate that their counterparts are found in other parts of the genome, probably as a result of translocation. Scale unit is Mbp. **c** For a detailed view of the paired structure, a 20 kbp region is magnified. Gene models (*yellow* or *pink*, *boxes* show exons and *arrows* show the gene orientation) and the histograms of RNA-seq coverage are shown under the gene models in both boxes. In the *lower box*, numbers of mismatches per 100 bp window (*cyan*) and lengths of insertions and deletions (*blue* and *red*, respectively, shown at 1 bp before In/Del site) are plotted in the 3rd and bottom rows, respectively. Scale unit is kbp

We performed a homology search among all the scaffolds. A Dot plot (Fig. 1a) shows that, in addition to a 100% match between selves (red lines), highly homologous regions (~94% similarity) are visualized as a series of purple lines that appear with an almost 45 degree slope by rearranging the order of scaffolds, suggesting a linear correlation between corresponding scaffolds. In other words, most of the contigs have homologous counterparts in our assembly. In fact, 89.3% of the scaffold sequences were covered by reciprocally best alignment segments. Thus, the genome consists of pairs of allelic sequences with a high degree of heterozygosity. Figure 1b shows a blow-up of a paired region: the homologous regions between the two scaffolds are aligned. Figure 1c shows a further blow-up of a part of the homologous region: in addition to the gene models and transcriptome (see below), the frequency of the single nucleotide variations (SNV) and short insertions and deletions (In/Dels) are depicted in the bottom part. The SNV frequency is variable but shows a tendency to be relatively high in intron regions. As shown in Table 1, the overall SNV frequency is 5.7% and In/Del 0.66%. The distribution of SNV ratios in individual CDS (coding sequences), introns and intergenic regions indicates that the occurrence of SNV is relatively uniform over the genome (Additional file 3). Inversions and translocations are also found (Additional file 4).

### Genome size and chromosome number

We measured the nuclear DNA content by flow cytometry to estimate the genome size using *C. elegans* (100 Mbp *×* 2/nucleus) and *D. melanogaster* (140 Mbp × 2/nucleus) as size references. These measurements indicate that the *D. coronatus* nuclear DNA comprises about 140 Mbp (Additional file 5). As this kind of measurement may contain considerable errors (up to ~25%) depending on the size standard used for the estimation [23, 24], the value of assembled 170 Mbp is within the error range. Microscopic measurements of fluorescently labeled nuclear DNA also indicated a similar value (Additional file 6). Thus, we conclude that our 170 Mbp assembly span closely reflects the actual genome size of *D. coronatus*.

**Fig. 2:**
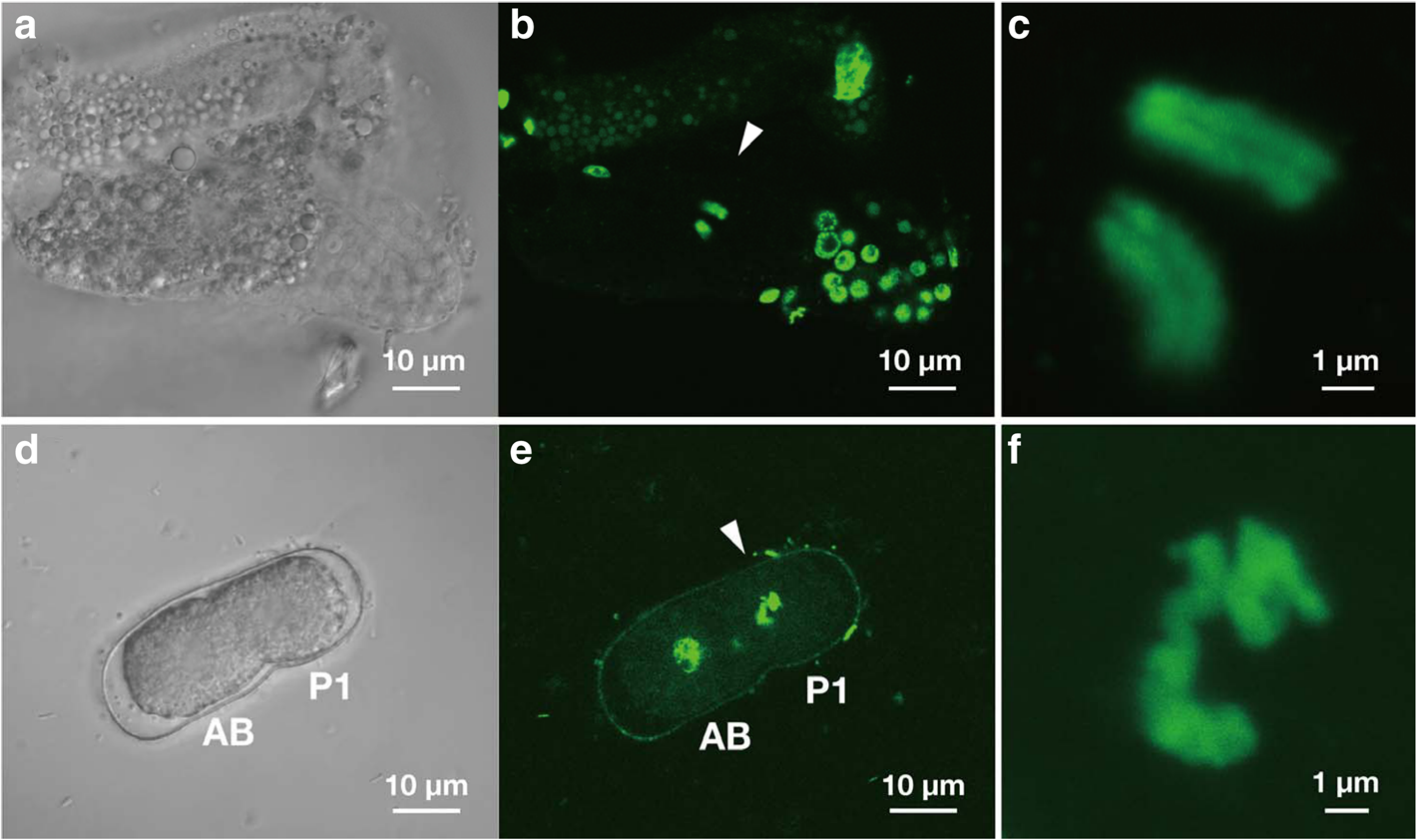
DAPI staining of *D. coronatus* chromosomes**. a**, **b** A gonad with a single uncleaved egg cell. **c** Magnification of the chromosomes in **b** (*arrowhead*). Note separation into chromatids. **d**, **e** A 2-cell embryo. **f** Magnification of the two condensed chromosomes in the P1 cell in **e** (*arrowhead*). **a**, **d** Nomarski images. **b**, **c**, **e**, **f** Fluorescent confocal microscopic images. Rotatable 3D images of c and f are given in Additional file 14

For karyotype analysis, we fluorescently marked chromosomes at two stages: late oocytes prior to the start of cleavage, and in early blastomeres (Fig. 2). At the same stage where in the *C. elegans* oocyte 4n = 24 chromatids can be detected (data not shown), we observed 4n = 4 chromatids in the *D. coronatus* oocyte (Fig. 2c). In early embryonic cells 12 chromosomes (2n = 12) were observed in *C. elegans*, but only 2 in *D. coronatus* (Fig. 2f). These results show that in the latter the diploid set consists of 2 chromosomes. This is in accordance with the earlier report of a different isolate by Hechler [25].

### Gene content of the parthenogenetic nematode *D. coronatus*

#### Repeat sequences and RNAs

Repeat sequences occupy 17.4% of the *D. coronatus* genome. In these repeats transposon-like sequences were identified and the number of these was comparable to *C. elegans* (Additional file 7: Table S5). We identified six 28S and five 18S rRNA genes. Two sets of these genes are found at the edges of long contigs and the others are in five short (< 9kbp) scaffolds. tRNA and other RNA families were also identified (Additional file 7: Table S3). Splice-leader sequences SL2 were found in addition to SL1 (Additional file 7: Table S4).

#### Protein coding genes

We obtained 140 million RNA-Seq reads from a mixed-stage population ranging from embryo to adult. Using Augustus [26, 27] with incorporation of the RNA-Seq data, we predict 34,421 protein coding genes, of which 58% are supported by our previously established EST library. Analysis with BUSCO [28] showed that the genome completeness was reasonable, taking into account the known imprecision of BUSCO for non-model organisms (Additional file 1: Table S2). Although 90 of the predicted genes showed homology only with non-metazoan sequences in the databases, we have no direct evidence for horizontally transferred genes in the *D. coronatus* genome.

Among the protein coding genes, 20,264 (59%) were shown to be orthologous to the *C. elegans* genes listed in WormPep [29] according to InParanoid [30, 31] (Table 2). Of these, 16,092 genes consisted of 8046 heterozygous pairs (doubletons), which were orthologous to a single *C. elegans* gene. They are considered as allelic partners encoded in the two “haploid” genomes present in our assembly. We call this phenomenon (Dc:Ce) 2:1 relationship (Additional file 8). The other 374 pairs (748 genes) show a 2:2+ relationship, where the *D. coronatus* genes are homologous to two or more *C. elegans* genes, suggesting a gene expansion in the *C. elegans* genome. Among the remaining genes, 3214 show various relationships like 3+:1 or 3+:2+ where more than three *D. coronatus* genes that are homologous to each other are homologous to one or more *C. elegans* genes. These are classified into 559 groups. The remaining 210 genes in the *D. coronatus* genome have no homologous partners and are assigned as singletons.

**Table 2:** Number of *D. coronatus* protein coding genes

Category^a^	genes		pairs/groups
Orthologs of *C. elegans* genes	20,264		
doubleton to a single *C. elegans* gene (2:1)		16,092	(8046 pairs)
doubleton to multiple *C. elegans* genes (2:2+)		748	(374 pairs)
gene family to *C. elegans* gene(s) (3+:1, 3+:2+)		3214	(559 groups)
singleton to *C. elegans* gene(s) (1:1+)		210	
Non-orthologs of *C. elegans* genes	14,157		
doubleton (2:0)		5850	(2925 pairs)
gene family (3+:0)		743	(191 groups)
singleton (1:0)		7564	
total protein coding genes	34,421		

^a^Relationships between genes of this category, e.g. (2:1) and (2:2+), are depicted in Additional file 8

Among the 14,157 (41%) protein coding genes that were not orthologous to *C. elegans* genes 5850 formed pairs. In addition, 743 genes in 191 groups formed triplets or more. The remaining 7564 genes were assigned as singletons (Table 2). The order of genes and their direction of transcription (co-linearity) are well conserved between the two allelic regions in the *D. coronatus* genome (see Fig. 1c). Thus, the counterparts of these singleton genes may have either been eliminated from the genome, or they might have been missed in our clustering analysis due to their high divergence or faulty gene predictions. Furthermore, there are still many contig gaps into which some counterparts might fall. Therefore, a closer examination of the sequences may identify more allelic counterparts.

Taken together, the predicted 34,421 genes are classified into 11,345 allelic pairs (22,690 genes), 7774 singletons and 750 groups of 3957 genes. It is difficult to define the gene number of the genome consisting of a pair of such diverged chromosomes. Under the simple assumption that all genes consist of allelic pairs except for singletons, the number of genes in the conventional diploid *D. coronatus* genome is 21,098 (=11,345 + 7774 + 3957/2). This number is close to what has been found in *C. elegans* [32]. It remains to be determined to what extent the paired genes may differ from each other in their function.

In parthenogenetic reproduction the allelic regions must have changed and evolved independently from each other. We calculated the base substitution ratio between the gene pairs that showed the 2:1 relationship in order to see whether there was any selection on the gene pairs. With respect to the CDS regions of the paired genes, the median identity is 97.2% in total, 93.7% at the 3rd letter, and 93.0% at the 4-fold degenerated site. This sequence divergence is similar to the diversity observed in *Caenorhabditis remanei* that shows one of the highest diversities in eukaryotes [33]. Of 8046 such pairs, 7306 pairs show size differences of less than 100 bp. We calculated dN/dS values (the ratio of substitution rates at non-synonymous and synonymous sites) of these pairs. Significant dN/dS values (*P* < 0.05) were obtained for 6760 pairs. The median of the values was 0.12 and none of them exceeded one, indicating that the majority of genes had diverged under negative selection and no sign of positive selection was detected.

### Allelic gene expression in *D. coronatus*

**Fig. 3:**
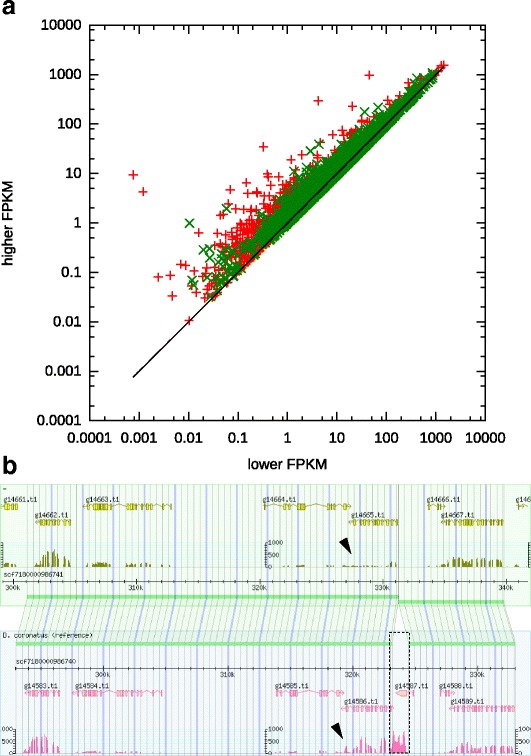
Allelic expression analysis. **a** Comparison between allelic gene expressions. The FPKM values for allelic pairs of *D. coronatus* genes, 7306 (*green cross*) with a single *C. elegans* ortholog and 2526 (*red cross*) without *C. elegans* ortholog, are plotted in such a way that the lower value is on the X-axis and the higher value on the Y-axis. **b** Region with 10-fold difference in the allelic expression level. A 40kbp region in the scaffold scf7180000986740 (*lower box*) and its homologous region in the scaffold scf7180000986741 (*upper box*) are shown with the gene models and the histograms of RNA-seq coverage (*pink* or *yellow*). The allelic pair genes g14665.t1 and g14586.t1 show a big difference in the RNA-seq pattern (*arrowheads*), while others show almost identical patterns. The gene g14587.t1 seems to be inserted just upstream of gene g14586.t1 (*dashed box*)

Heterozygosity between the allelic genes is so high that even short sequences like 100 bp RNA-Seq reads can be assigned to either of the allelic sequences. This allowed us to analyze the expression level of individual alleles (“allelic expression analysis”). As shown in Figs. 1c and 3a, in spite of the high sequence divergence which must cause changes in regulatory sequences, most of the homologous pairs show very similar expression levels: among the 7306 gene pairs, FPKM (fragments per kilo bases of transcript per million fragments sequenced) ratio for 6736 pairs is less than 1.5-fold and the correlation coefficient of FPKM is 0.99. However, some pairs show deviant expression levels: 121 pairs show >2-fold difference, and 5 pairs show >5-fold difference (Fig. 3a). For example, between the orthologous genes of K03H6.1 (G-protein-coupled receptor), the expression level of one allele, g14586.t1, is 10-fold higher than the other allele, g14665.t1 (Fig. 3b). In this case, an insertion (or deletion at the opposite site) of the gene g14587.t1, which has the mariner transposase-like sequence, is found 370 bp upstream of the translational initiation codon of g14586.t1. This insertion might cause the increase of transcription. We searched the genome for this transposon-like sequence and found 54 loci. In one case, it is inserted in an intron and an increase of the downstream transcription is observed. These differentially expressed genes are dispersed and not clustered in the genome.

### Peculiarities in the gene repertoire of a parthenogenetic nematode

We examined the presence or absence of *D. coronatus* orthologs of *C. elegans* genes for core biological processes [29, 34] by InParanoid analysis. In the following sections, we summarize the results focusing on genes related to the mode of reproduction (See Additional file 9 for others).

#### Genes involved in sex determination

In *C. elegans*, sex is determined by the X chromosome to autosome (X/A) ratio, which is read by chromosomal counting factors that regulate gene expression in the sex-determination cascade, i.e. *xol-1* and dosage compensation (*sdc*) genes [35–37]. The sex-determination signal is transmitted to individual cells through *her-1*/*tra-2* ligand-receptor genes [38–40]. At the end, the terminal transcription factor TRA-1 regulates all aspects of hermaphrodite sexual differentiation in somatic cells.

In *D. coronatus,* a number of key components in this pathway e.g. *xol-1*, *tra-2*, were missing in our search (Additional file 10). It may not be surprising that a parthenogenetic species lost genes in the sex determination pathway. However, it is interesting that a considerable number of orthologs are retained in this pathway, i.e. SEX-1 and FOX-1 (X chromosome counting factors), and HER-1 (TRA-2 ligand). In *D. coronatus,* they might function in a pathway other than the original sex determination pathway.

#### Genes involved in meiosis

In *C. elegans*, sister chromatid cohesion is established during DNA replication [41, 42] by a cohesin complex that contains the meiosis-specific kleisins, REC-8 and COH-3/4. The pairing of homologous chromosomes is initiated at the pairing center by recruiting the chromosome-specific zinc-finger proteins ZIM-1/2/3 and HIM-8 [43–46]. These proteins anchor chromosomes to the nuclear envelope through binding to SUN-1 and ZYG-12 proteins present there. This process facilitates homologous chromosome pairing [47]. To form the synaptonemal complex (SC), HTP-3 localizes on the chromosomes and recruits the other axial element components HIM-3, HTP-1/2 and the transverse filaments SYP-1/2/3/4 [41, 48]. Then, meiotic recombination takes place triggered by DNA double-strand breaks via SPO-11, followed by strand invasion mediated by RAD-51 and RAD-54 [41].

**Fig. 4:**
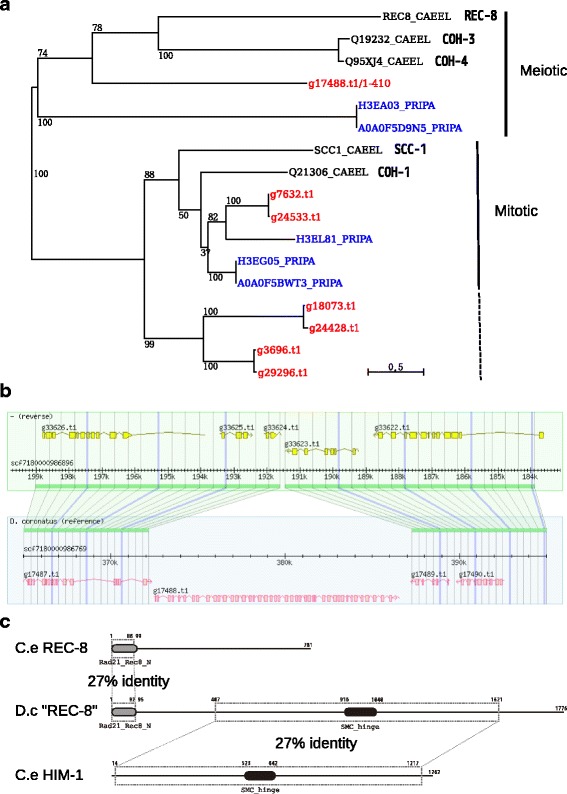
Analysis of REC-8 homologs. **a** Maximum likelihood unrooted phylogenetic tree of the amino acid sequences of the REC-8 homologs in *D. coronatus* (*red*), *C. elegans* (*black*; canonical gene names are shown in addition to UniProt IDs) and *P. pacificus* (*blue*). Numbers are bootstrap values in percent. Scale bar indicates 0.5 replacements/site. One meiotic kleisin (g17488.t1; 410 residues of the N-terminus were used in the analysis) and three pairs of mitotic kleisins can be identified in *D. coronatus.* **b** Genomic structure (30 kbp) around the putative *D. coronatus* REC-8 (g17488.t1) and its allelic partner. The gene is present only in one allelic partner. **c** Protein structure of the putative D.c (*D. coronatus*) REC-8. Similarities with C.e (*C. elegans*) REC-8 and HIM-1 are shown. Numbers indicate positions in the amino acid sequences. The homologous regions are marked by *dotted boxes*. Some Pfam domains are indicated by *ovals*. The putative D.c REC-8 seems to be a fusion of the N-terminus of REC-8 and the complete HIM-1

Screening our *D. coronatus* genome, we could not detect credible orthologs of many key genes in meiotic development [49], such as, *rec-8, coh-3/4, zim-1/2/3, him-8,* and *syp-1/2/3/4* by InParanoid analysis (Table 3). We thus further searched for homologs of these genes using the Pfam database [50], and found four kleisin homologs (three allelic pairs and a singleton) in the *D. coronatus* genome. According to our phylogenetic analysis, *D. coronatus* possesses three alleles of mitotic kleisin (SCC-1/COH-1) homologs and one singleton of the meiotic kleisin (REC-8/COH-3/4) homolog (Fig. 4a). The meiotic kleisin homolog, g17488.t1 (D.c “REC-8”), however, shows atypical features: (1) It does not have an allelic counterpart in the genome while all three mitotic kleisins are present as allelic pairs (Fig. 4b), (2) It contains only the N-terminal domain of REC-8 and fuses with HIM-1(SMC-1), an interacting structural protein of kleisin in the cohesin complex (Fig. 4c), (3) It lacks the C-terminal domain of kleisin known as the Rad21/Rec8-like domain C-terminal (IPR023093). Usually, the N-terminal and C-terminal domains of kleisins interact with SMC-3 and HIM-1(SMC-1), respectively. However, in *D. coronatus* the REC-8 homolog has lost its C-terminal domain for SMC-1 binding and instead is fused directly to SMC-1. The meiotic kleisins are essential factors to hold sister chromatids together during meiosis, thus, their absence or divergence may well be related to parthenogenetic reproduction. Phylogenetic analysis of other meiosis-specific genes showed that they have orthologous counterparts in the *C elegans* genome mostly in pairs (Additional file 11).

**Table 3:** Meiosis-related genes in *D. coronatus*

Gene (alias)^a^	Presence^b^	Homolog/ Ortholog^c^	Gene (alias)^a^	Presence^b^	Homolog/ Ortholog^c^
*air-2*	+	Aurora/IPL Kinase	*mes-4*	+	WHSC1; SETDB1
*aspm-1*	+	ASPM	*met-1*	+	SETD2
*atm-1*	−	ATM	*met-2*	+	SETDB1
*brc-1*	+	BRCA1	*mix-1*	+	SMC2
*brc-2*	−	BRCA2	*mpk-1*	+	ERK
*capg-1*	+	hCAP-G	*mre-11*	+	MRE11A
*capg-2*	+	hCAP-G2	*mrg-1*	+	Chromodomain protein
*cep-1*	−	p53	*mrt-2*	+	RAD1
*chk-2*	+	CHK2	*msh-4* (*him-14*)	+	MSH4
*cls-2*	+	CLASP	*msh-5*	+	MSH5
*cmd-1*	+	CALM1	*mus-81*	+	MUS81
*coh-3*	−	Rad21/Rec8	*pch-2*	+	TRIP13
*coh-4*	−	Rad21/Rec8	*plk-1*	+	PLK1
*com-1*	−	CtlP / Sae2	*plk-2*	+	PLK1
*cosa-1*	+	CTND1	*pph-4.1*	+	PPP4C
*cra-1*	+	NAA25	*prom-1*	+	FBXO47
*cye-1*	+	CCNE1	*rad-50*	+	RAD50
*dpy-26*	+	hCAP-H	*rad-51*	+	Rad51; DMC1
*dpy-28*	+	hCAP-D2	*rad-54*	+	Rad54
*dsb-1*	−		*rec-1*	−	
*dsb-2*	−		*rec-8*	−	Rec8
*egl-1*	−		*rfs-1*	+	RAD51C
*exo-1*	+	exo1	*rpa-1*	+	RPA1
*fcd-2*	−	FANCD2	*rtel-1*	+	RTEL1
*gsp-2*	+	PP1CA	*scc-2* (*pqn-85*)	+	SCC2
*hal-2*	−		*scc-3*	+	SCC3
*hcp-6*	+	hCAP-D3	*slx-1*	+	SLX1
*helq-1* (*hel-308*)	+	HELQ	*smc-1* (*him-1*)	+	SMC1
*him-3*	−	HORMAD1; Hop1	*smc-3*	+	SMC3
*him-5*	−		*smc-4*	+	SMC4
*him-6*	+	BLM; RecQ	*smc-5*	+	SMC5
*him-8*	−		*smc-6*	+	SMC6
*him-17*	−		*spd-3*	+	
*him-18*	+	SLX4	*spo-11*	+	SPO11
*him-19*	+		*sun-1*	+	SUN-domain family
*hpr-9*	+	RAD9	*syp-1*	−	
*htp-1*	+	HORMAD1; Hop1	*syp-2*	−	
*htp-2*	+	HORMAD1; Hop1	*syp-3*	−	
*htp-3*	−	HORMAD1; Hop1	*syp-4*	−	
*hus-1*	+	HUS1	*tim-1*	+	TIMELESS
*kle-2*	+	hCAP-H2	*unc-116*	+	Kinesin 1
*klp-18*	+		*xnd-1*	−	
*klp-19*	+	Chromokinesin	*xpf-1*	+	ERCC4
*lab-1*	−		*zhp-3*	+	RNF212; Zip3
*lin-5*	+	NuMA	*zim-1*	−	
*lin-41*	+		*zim-2*	−	
*mei-1*	+	KATNAL1	*zim-3*	−	
*mei-2*	−		*zyg-12*	+	KASH-domain family

^a^Meiosis-related genes in *C. elegans* (modified from [49])

^b^Presence (+) or absence (−) of their *D. coronatus* homologs based on InParanoid analysis

^c^Names of the respective orthologous or homologous genes in other organisms

The pairing center recognition proteins, ZIM-1/2/3 and HIM-8 are absent in *D. coronatus,* but their interacting proteins SUN-1 and ZYG-12 are present (Table 3), suggesting that unknown proteins may mediate chromosome-nuclear membrane interaction. Although SC proteins are evolutionarily variable and share only a low similarity [51], the obvious loss of the synaptonemal complex (SC) components SYP-1/2/3/4, may not be compatible with homologous chromosome pairing in *D. coronatus.*

## Discussion

In the present work, we have analyzed the genome of the parthenogenetic nematode *Diploscapter coronatus*. A central question related to parthenogenesis is how such organisms are nevertheless able to preserve the necessary diversity of the gene pool. *D. coronatus* appears to be a good system to elucidate the molecular idiosyncrasies of parthenogenesis as it is a close relative of the well-studied hermaphroditic model organism *C. elegans*. A genome comparison between these two species should provide clues to the genetic basis of different reproductive modes.

Our analysis showed that the *D. coronatus* genome possesses a high degree of heterozygosity or allelic divergence. Genomes of organisms collected from the wild are often difficult to sequence due to their heterozygosity. This is because the genome assembly algorithm recognizes heterozygous regions as branch structures, leading to the termination of contig extension. Thus, some researchers resorted to inbred lines. The potato genome project used offspring produced by parthenogenesis (containing only the haploid genome of the mother) to avoid the heterozygosity problem [52]. Alternatively, various assembly programs including PLATANUS have been developed to overcome the problem with wild-derived organisms [53]. In *D. coronatus,* however, we were able to successfully assemble the shotgun reads into 170 Mbp sequences using a conventional assembly software. This is probably because this genome is so heterozygous that the genome assembler recognizes the allelic regions as separate sequences. The distribution of sequence read coverage shows a normal distribution (Additional file 12), meaning that rarely two different regions are assembled together because of nearly identical sequences. However, this means, in turn, that less heterozygous or nearly identical sequences, e.g. rRNA genes, result in a branched structure and thus it becomes difficult to assemble them into long contigs/scaffolds; indeed, in our assembly all rRNA genes are located at the end of contigs. Therefore, we plan to analyze the genome structure on a larger scale, hopefully from telomere to telomere, by using a new approach such as the Irys technology [54].

As mentioned above, 89% of the 170 Mbp assembled sequences can be aligned in pairs (Fig. 1). These homologous paired sequences are on average 94.3% identical (5.7% heterozygous) at the nucleotide level, and, if focused on CDS, the identity is 97.2%. This value is comparable to that of the bdelloid rotifer *Adineta vaga* (96.2%) [7], which reproduces as a constitutive mitotic parthenogen [21]. The genome of this rotifer is degenerate tetraploid with allelic pairs sometimes found on the same chromosome, referred to as permanent translocation heterozygosity (PTH), preventing meiosis [8, 10, 55]. However, such a peculiarity is not found in the *D. coronatus* genome, raising the question of how this organism carries out parthenogenetic reproduction.

Normally, during meiosis I, homologous chromosome separation takes place and the primary oocyte divides into two daughter cells (the secondary oocyte and the first polar body), each carrying two identical sister chromatids. Thus, if meiosis II were suppressed like in *D. coronatus,* even if crossing-over would take place, homozygosity would be preserved. However, we found that its genome exhibits an extraordinary high degree of heterozygosity, which corresponds to the level called “hyperdiversity” [56]. A look at the process of sister chromatid separation may help to solve the apparent contradiction.

In *C. elegans*, this critical event requires the function of the meiotic kleisins, REC-8, COH-3 and COH-4. These proteins tether sister chromatids together to assure proper separation of homologous chromosomes during meiosis I [42]. The loss of all three meiotic kleisins (REC-8, COH-3 and COH-4) results in premature sister chromatid separation during the first meiotic division and subsequent inhibition of meiosis II [57]. Our analysis revealed that there are no meiotic kleisin orthologs in the *D. coronatus* genome other than a single atypical one. Phylogenetic analysis revealed that *D. coronatus* possesses three pairs of mitotic kleisin homologs, however, two are located in a different branch compared to the mitotic kleisin “ortholog (g7632.t1/g24533.t1)” (Fig. 4a). Although branch lengths indicate that these genes belong to the mitotic kleisins, we cannot exclude that they may take over a function in meiosis. The atypical homolog lacks the C-terminal domain of REC-8 and contains only the N-terminal domain that is directly fused to HIM-1 (SMC-1) homolog. The function of this atypical homolog is not known, but it may result in a similarly modified meiosis I as in the manipulated *C. elegans* [57]. Another possible mechanism is the so-called “inverted meiosis” where sister chromatid separation occurs during meiosis I. This phenomenon is found under natural conditions in diverse animals and plants with holocentric chromosomes [58, 59]. Such organisms face a specific kinetochore geometry problem, for which inverted meiosis is a possible solution. *C. elegans* and all studied members of neighboring nematode clades also possess holocentric chromosomes, nevertheless *C. elegans* follows the conventional meiotic order [60–63]. It remains to be tested whether *D. coronatus* makes use of this inverted meiosis.

It has been claimed that parthenogenesis commonly arises via interspecies hybridization [5]. The plant-parasitic nematode *Meloidogyne incognita*, which reproduces by obligate mitotic parthenogenesis, is thought to originate from such a hybridization event [15]. A comparative genome analysis of three *Meloidogyne* nematodes, *M. incognita, M. floridensis* and *M. hapla,* revealed the complex hybrid origin of the *M. floridensis* [14]. Some of the *M. floridensis* and *M. incognita* genome features are similar to those observed in *D. coronatus*. More than half (64%: 55 Mbp / 86 Mbp) of the *M. incoginita* genome consists of genomic regions in two copies [12], (*D. coronatus*: 89%: 152 Mbp / 170 Mbp). The nucleotide divergence between the pairs is 8% in *M. incognita* and 5.7% in *D. coronatus*. The *M. incognita* genome has large duplicated and rearranged regions, which may restrict recombination of chromosomes, while translocations and inversions are observed in the *D. coronatus* genome (Additional file 4). However, there are differences, too. In *M. incognita* no meiosis occurs during the production of the female gamete and the eggs are derived from unreduced oocytes by mitotic cell division, whereas *D. coronatus* executes meiosis, albeit truncated. Transposable elements and repetitive sequences, which are hypothesized to be related to the asexual mode of reproduction, comprise 36% of the *M. incognita* genome but only 17.5% of the *D. coronatus* genome (Additional file 7: Table S5). The latter value is similar to that in nematodes showing sexual reproduction (16.5%: *C. elegans*, 22.4%: *C. briggsae*) [6, 64]. These data suggest that the mechanism of how parthenogenesis was acquired differs between these two species.

In the *D. coronatus* genome, nearly 90% of the assembled sequences that have paired structure show good co-linearity over a long range, although with many inversions and translocations (Additional file 4). It is also remarkable that expression levels are extremely similar between the allelic genes despite the high heterozygosity of the *D. coronatus* genome. If *D. coronatus* was the product of an interspecies hybridization, it must have taken place between very close relatives. Therefore, we have started to search for close relatives of this species with bisexual reproduction as potential parent species of our strain. So far, we only found representatives of the neighboring genus *Protorhabditis*. With respect to chromosomes, two of them are like *D. coronatus* (2n = 2) while another one is like *C. elegans* (2n = 12) [20]. Alternatively, whole genome duplication (WGD) followed by diversification of the gene duplicates (ohnologs) could have led to a similar situation as after interspecies hybridization. Finally, a mechanism called “Meselson effect”, i.e. an independent accumulation of mutations, inversions and translocations as a consequence of parthenogenetic reproduction could be responsible for the observed diversity of the gene pool [1, 21] . Our current data do not allow us to determine the origin of heterozygosity in *D. coronatus,* however, the dN/dS ratio might give a clue. If *D. coronatus* is a result of hybridization, the considerable divergence between the two gene copies would probably indicate the original divergence between the two parent species and thus should have a strong signature of negative selection. In the WGD model, the ohnologs should acquire deleterious mutations resulting in a relatively high dN/dS ratio. The same should be true when a Meselson effect applies. The dN/dS ratio in *D. coronatus* did not exceed one and the median was 0.12, indicating negative selection. Thus, these data appear to be in favor of a hybridization origin. In any case, the genome analyzed in this work provides a solid basis to further explore the mechanism of parthenogenesis and the evolution of nematode diversity.

## Conclusions

Our high-quality draft genome of *D. coronatus* reveals the genome peculiarities of a parthenogenetic nematode. We obtained a 170 Mbp draft genome in only 511 scaffolds with a N50 length of 1 Mbp. Nearly 90% of these scaffolds constitute homologous pairs with a 5.7% heterozygosity together with many inversions and translocations, and most of the genes exist in two distinct alleles. These features mean that the 170 Mbp sequences correspond to the diploid genome. DAPI staining shows that the *D. coronatus* genome consists of two chromosomes (2n = 2). The high degree of heterozygosity allowed us to analyze the expression level of individual alleles. Most of the homologous pairs show very similar expression levels but others exhibit a 2–5-fold difference.

The draft genome provides some clues to the genetic basis for parthenogenetic reproduction. In our genome annotation, we found orthologs of 59% of the *C. elegans* genes. However, a number of genes were missing or very divergent. These include genes involved in sex determination (e.g. *xol-1, tra-2*) and meiosis (e.g. the kleisins *rec-8* and *coh-3/4*) giving a possible explanation for the absence of males and the second meiotic division.

This draft genome constitutes a solid basis for the elucidation of fundamental questions related to parthenogenesis such as its origin and underlying mechanisms in conjunction with comparative analyses of other nematodes. Furthermore, being the closest outgroup to the genus *Caenorhabditis*, our draft genome can help to disclose many idiosyncrasies of the model *C. elegans* and its congeners in future studies.

## Methods

### Strain and culture

*Diploscapter coronatus* strain PDL0010 was originally obtained from Prof. P. De Ley, Dept. of Nematology, University of California, Riverside and has been maintained in the Schierenberg laboratory [16]. The strain was cultured at 20 °C on the standard NGM agarose plates that were seeded with the OP50 strain of *Escherichia coli* as a food source [65] and covered with a thin layer of distilled water to prevent the nematodes from digging into the agar.

### DNA and RNA preparation

*D. coronatus* were washed off the agar plates and collected on 10 μm-mesh nylon filters. The nematodes were transferred to a 1-l flask containing 100 ml of distilled water and incubated for 2 h to allow digestion of remaining food bacteria. Nematodes were collected by filtration, aliquoted ~200 mg into 2.2 ml tubes and stored at -80 °C. 200–400 mg of packed worms were ground in a mortar in liquid nitrogen and used for a single DNA/RNA preparation. Genomic DNA was purified with the Genomic-tip 500/G Kit, according to the manufacturer’s instructions (Qiagen, Hilden, Germany). RNA was purified by RNAgents Total RNA Isolation System (Promega, Fitchburg, WI, USA) and polyadenylated RNA was purified with a mRNA Purification Kit (GE Healthcare Life Sciences, Buckinghamshire, UK) using an Oligo(dT)-cellulose column.

### Library construction and sequencing for genomic DNA

Sanger sequencing was performed as described [66]. Briefly, for shotgun libraries, *D. coronatus* DNA was sheared randomly by Hydroshere (DIGILAB, Marlborough, MA, USA), and then the sheared DNA was end-repaired, phosphorylated and ligated into the SmaI site of pUC18 with the TaKaRa BKL Kit (Takara, Shiga, Japan). The ligated samples were purified by phenol extraction and transformed into *E. coli* DH5α by electroporation. Sequencing reactions were performed with BigDye terminator cycle sequencing kits using the M13F and M13R primers, and run on an ABI 3730xl analyzer (Applied Biosystems, Foster City, CA, USA). For fosmid sequencing, *D. coronatus* genomic DNA was randomly sheared by pipetting, and the DNA was polished and dephosphorylated by Mung Bean Nuclease, T4 DNA polymerase and alkaline phosphatase (NEB, Ipswich, MA, USA). The DNA was ligated into a pKS300 fosmid vector and packaging reactions were performed using Giga Pack III XL packaging extract (Stratagene/Agilent, Santa Clara, USA). The packaged fosmid library was transfected into *E. coli* XL1-Blue. Clones were picked randomly and sequenced in the same way as for shotgun analysis.

Next generation sequencing (NGS) was performed as described [67]. Briefly, sequencing libraries were prepared using the GS FLX Titanium Rapid Library Preparation Kit (F. Hoffmann-La Roche, Basel, Switzerland) and the TruSeq DNA Sample Prep Kit (Illumina, San Diego, USA), and these libraries were run on a GS FLX and a Miseq sequencer, respectively.

### Library construction for transcriptome analysis

cDNA libraries were generated by three different full-length enriched cDNA construction methods. (1) The NDK cDNA library was prepared using the Creator SMART cDNA library construction kit with the pDNR-LIB vector (Clontech/Takara, Shiga, Japan), according to the manufacturer’s protocol. (2) The NDF library was prepared by the oligo-capping method using the pME18S-FL3 vector [68]. (3) The NDV library was constructed by the vector-capping method [69] using the pGCAP10 vector (Hitachi High-Tech and Hokkaido System Science, Japan).

The RNA-Seq library was prepared with the RNA-Seq Sample Prep Kit according to the manufacturer’s instructions (Illumina, USA).

### Quantification of nuclear DNA by flow cytometry

*D. coronatus* and *C. elegans* (genome size: 100 Mbp) were washed out and collected with a 10 μm nylon filter. Nematodes were transferred to a 300 ml flask containing 50 ml of distilled water and incubated for 60 min to reduce ingested food bacteria. Five head parts of *Drosophila melanogaster* were also prepared as the standard for genome size (140 Mbp). Worms and fly heads were homogenized in sodium citrate buffer (pH 7) using a Dounce homogenizer by hand for 10 strokes. The homogenate was centrifuged at 400×g for 3 min to remove debris. The supernatants were treated with trypsin in a spermine tetrahydrochloride detergent buffer and stained with 125μg/ml propidium iodide (PI) (for details, see Cycle TEST PLUS DNA Reagent Kit manual (BD Biosciences, Franklin Lakes, NJ, USA)). The standards and *D. coronatus* samples were analyzed individually, and their mixture was analyzed by flow cytometry. Flow cytometry was performed with a Desktop cell sorter JSAN (Bay bioscience, Tokyo, Japan).

### Chromosome staining

Adult *D. coronatus* were transferred to a drop of M9 buffer [65] containing 25 μM levamisole and 0.1 μg/ml 4′,6-diamidino-2-phenylindole dihydrochloride (DAPI), and the gonad was dissected by nicking with a scalpel blade behind the pharynx. The slides were frozen on dry ice, and thawed at room temperature before microscopic observation. Images were recorded and analyzed with FV1200 confocal microscope using 100× UPlanSApo objective (Olympus, Tokyo, Japan). *C. elegans* worms were examined as a control with the same protocol. We performed a closer inspection of 11 oocytes (4n was observed in two oocytes, 2n was in six) and 6 embryos (2n was in three).

### Data analysis

Data processing was done in the NIG supercomputer facility [70] using BioPerl [71] (version 1.6.1), EMBOSS [72] (version 6.4), BEDtools [73] (version 2.16.2), SAMtools [74] (version 0.1.18) and the other programs described below, which are installed in the super computer system as standard software. The sequence data were assigned BioProject accession PRJDB3143.

### EST clustering

This was carried out by an in-house UNIX shell script with a short program written in C (Additional file 13). Briefly, first we take one clone and compare its 3′ end sequence with the 2nd clone using the FASTA program. If there is a match above a threshold (usually 90% considering the errors in EST sequencing), they are grouped, and if not, they are assigned a different group. The 3rd clone is compared with the previous ones, and, if there is match, it is included in the existing group, and if not, it is assigned a new group. Repeating this process, we classify EST clones based on the 3′ end sequences.

### Genome assembly

The genome sequence was assembled from all the four libraries together by the Celera assembler [22] (options: “gkpFixInsertSizes = 0 bogBadMateDepth = 1000 cgwDemoteRBP = 0” and “doTrim_initialMerBased = 0 doTrim_initialQualityBased = 1” for Illumina Miseq reads, version: 7.0). The obtained sequences were 177,655,898 bp in 971 scaffolds consisting of 1817 contigs and 12,242,269 bp in 38,996 degenerate (meaning unused repeats) contigs. The statistics of the reads used (trimmed in the assembly process) are found in Additional file 1: Table S1. Miseq reads were re-mapped to the scaffolds and degenerates by BWA [75] (version 0.6.1-r104). Based on the results, 520 scaffolds, which were mapped at more than 0.01 reads/bp and longer than 2 kbp, were selected. A long scaffold apparently derived from the food bacterium *E. coli* OP50 was thus discarded at this stage. Furthermore, nine scaffolds turned out to represent the mitochondrial genome as described below. As a result, the remaining 511 scaffolds were considered to represent the *D. coronatus* genome. All analyses were performed with these.

The mitochondrial genome sequence was assembled manually using Consed [76] (version 29, with phrap version 1.090518) with the reads collected from the shotgun Sanger sequencing library based on the homology to the mitochondrial genome sequence of *C. elegans* [77]. A circular genome sequence of 13,378 bp was obtained. Covariance models for 22 tRNA genes were built from the alignments of Nematoda tRNA sequences in the mitotRNAdb database [78] and were searched for by Infernal [79] (version 1.1rc2). rRNA and protein coding genes were identified, such that the ranges on the whole genome alignment were similar to the *C. elegans* genes, assuming TTT as a start codon and T (with polyadenylation after transcription) as a stop codon [80, 81].

### Paired structure of the genome

At first, the MUMmer package [82] (version 3.23) was used for the whole genome alignment. The scaffold sequences were aligned by nucmer (options --maxmatch --nosimplify). Trivial hits (alignments to themselves) were removed, delta-filter (option −1) was applied, and the scaffolds were reordered by mummerplot (option --fat) such that the resultant 1-to-1 alignments were emphasized by placing them diagonally on a Dot plot. In parallel, the alignments of minimal sequence identity 90% were filtered by delta-filter (option -i 90) from the nucmer result. Figure 1a is the plot of the >90% identity alignments with the order emphasizing the 1-to-1 alignments.

Next, longer alignments were obtained by the LAST package [83] (version 460). The scaffold sequences without masking were aligned by lastal (option -e1000). After trivial hits had been removed, the reciprocally best alignment segments were obtained by applying last-split (option -m 1) twice with maf-swap.py in-between. The alignments covered 152,151,424 bp (89.3% of the genome) and 8,685,973 bp (5.7% of them) were mismatches. To compare the partners of the alignments visually, the Syntenic Browser of GBrowse [84, 85] (version 2.55) was set up [86]. The length of each gap on the alignments and the number of mismatches on each 100 bp window were counted and loaded onto the browser in addition to the annotations (Fig. 1c).

### Repeat contents

Repeat sequences were identified de novo in the 971 scaffolds (before cleaned up) by RepeatModeler [87] (version 1.0.7 with RepeatScout version 1.0.5, RECON version 1.07). The obtained 754 repeat sequences were used by RepeatMasker [88] (options: -s -gccalc, version: 4.0.1 with RMBlast version 2.2.27, HMMER version 3.1-snap20121016.1 and TRF version 4.0.4) and 17.4% of the 511 scaffolds (after cleaned up) were masked by the de novo modeled repeats or simple repeats. The 754 repeat sequences were analyzed by REPCLASS [89] (version 1.0.1 with RepBase version 22.03, blast version 2.3.0, options for tblastx: -evalue 0.0001 -num_descriptions 10,000,000 -num_alignments 10,000,000 -seg yes and options for blastn: -task blastn -gapopen 2 -gapextend 1 -reward 1 -penalty −3 -dust no). 423 out of the 754 sequences, which was longer than 100 bp and whose copy number in the genome was greater than nine, were subjected to the classification procedure.

### RNA genes

tRNA and rRNA genes were predicted by tRNAscan-SE [90] (version 1.23) and RNAmmer [91] (version 1.2 with HMMER version 2.3.2) respectively. RNA families in the Rfam database [92] (release 11.0) were searched by Infernal.

### Protein coding genes

From the paired-end reads of the RNA-seq library, the adapter sequences were removed by SeqPrep [93] (version 1.1). Because almost all (92.8%) of the paired end reads could be merged to single sequences by this process, we used only the merged sequences. The merged reads were mapped to the genome sequence by TopHat [94] (options: --min-intron-length 5 --min-segment-intron 5, version: 2.0.5 with bowtie version 2.0.0-beta7). 91% of the reads could be mapped and 88% of the mapped reads were uniquely mapped.

Protein coding genes were predicted by Augustus [26, 27] (options: --species = caenorhabditis --allow_hinted_splicesites = atac --alternatives-from-evidence = false --min_intron_len = 8, version: 2.7) using the hints from the mapping result of the RNA-seq (bam2hints with options --intronsonly --maxgaplen = 0 --minintronlen = 8 --maxintronlen = 10,000, bam2wig and wig2hints.pl with options --width = 10 --margin = 10 --minthresh = 2 --minscore = 4 --prune = 0.1 --radius = 4.5 were used to prepare the hints and the configuration file “extrinsic.M.RM.E.W.cfg” in the Augustus package was applied). 33,459 genes were obtained.

Additional genes were predicted from the mapping result of RNA-seq by Cufflinks [95, 96] (options: --min-intron-length 5 --max-intron-length 25,000 --overlap-radius 5, version 2.0.0). If the prediction was placed intergenic of the Augustus predicted genes (class_code “u” was assigned by cuffcompare) and its longest open reading frame was longer than 89 bp, the model was adopted. 962 genes were obtained in this way. Together with the Augustus predicted genes, 34,421 protein coding genes were predicted.

### Gene expression levels

The expression levels of the total 34,421 gene models were estimated again by Cufflinks without predicting new isoforms (options -G -b -u).

EST sequences were cleaned up by SeqClean [97] (option -v, version x86_64) using spliced leader (SL) sequences of Nematoda [98]. The sequences removed of poly-A and SL were mapped to the genome by exonerate [99] (options -m est2genome -bestn 1, version 2.2.0). The coding sequences of 20,003 genes were overlapped with (or supported by) the EST mapping.

### Homology analyses

Protein categories were predicted by InterProScan [100] (option -goterms, version 5.3-46.0 with PANTHER version 8.1 data and Phobius version 1.01). 5859 InterPro entries were assigned to 20,264 genes.

The homologous (orthologous, in the conventional sense) gene groups between *D. coronatus* and *C. elegans* were obtained by InParanoid [30, 31] (options: seq_overlap_cutoff = 0 segment_overlap_cutoff = 0, version: 4.1 with BLAST version 2.2.26). The longest isoforms of 20,520 *C. elegans* genes (version wormpep230 [29]) were used in the analysis. As a result, 9189 homologous groups consisting of 20,264 genes of *D. coronatus* and 11,003 genes of *C. elegans* were obtained. To estimate total gene number, the remaining protein sequences of *D. coronatus* were clustered by cd-hit [101, 102] (option -g 1, version 4.6.1) with a threshold identity of 90%.

The coding sequences of the gene pairs of Dc:Ce = 2:1 were aligned by prank [103](option -codon, version 140110) and dN/dS were calculated by KaKs Calculator [104](option -m MLWL, version 1.2).

From the *D. coronatus* genes which belong to the orthologous groups of Dc:Ce = 2:1, 7306 pairs of genes, whose predicted CDS lengths differ by less than 100 bp, were selected as “allelic pairs”. The expression levels of the paired genes, indicated by their FPKM values, were compared and Pearson’s correlation coefficient of the higher and lower FPKM values was calculated (*P* < 2.2e-16).

### Search for REC-8 homolog

The domain model of the N-terminus of the Rad21/Rec8 like protein, Rad21_Rec8_N, was retrieved from the Pfam database [50] (release 27) and the proteins of *D. coronatus* were searched by hmmsearch [105] (option --max, version 3.1b1). The proteins of *C. elegans* and *P. pacificus* were retrieved from the UniProt database [106] by the query expression ‘database:(type:pfam Rad21_Rec8_N) AND (organism:6239 OR organism:54,126)’. The sequences were aligned by MAFFT [107] (option --linsi, version 6.864b), the alignment was trimmed by trimAl [108] (option -automated1, version 1.2rev59) and the maximum likelihood unrooted tree with bootstrap values was constructed by RAxML [109] (options -f a -m PROTGAMMAAUTO -N autoMRE, version 8.1.17). The amino acid substitution model LG [110] with empirical amino acid frequencies and 200 replicates for bootstrapping were assigned. The phylogenetic tree was drawn by SeaView [111] (version 4.4.2).

Low complexity regions of the *D. coronatus* proteins were masked by segmasker [112] (version blast 2.2.25). The *D. coronatus* proteins were searched for the *C. elegans* REC-8 protein by ssearch [113] (options -s BP62 -S, version 36.3.5c) . The N-terminus of *C. elegans* REC-8 and the *D. coronatus* homolog could be aligned but the score was quite insignificant (E-value 4.8). The *C. elegans* HIM-1 protein could be aligned to the *D. coronatus* REC-8 homolog with high significance, though the rank in the search was third (the top and second hits formed a 2:1 group with *C. elegans* HIM-1 in the InParanoid analysis).

## Additional files

Additional file 1: Table S1. Sequence reads for analysis. The numbers of genome shotgun reads, RNA-seq reads and ESTs are shown. **Table S2.** Genome assembly statistics. Information on genome assembly, paired regions between scaffolds, SNVs in the paired regions, In/Dels in the paired regions and BUSCO assessment results are shown. (DOCX 36 kb)

Additional file 2: k-mer distribution analysis. k-mer distribution in the Miseq library was analyzed with kmerspectrumanalyzer (version b584039 with jellyfish version 2.0.0, numpy version 1.8.1 and scipy version 0.12.0) [114]. The frequency of each 21-mer in the library was measured and the number of distinct 21-mers for each frequency is plotted (red cross). This frequency spectrum was fitted as a mixture of over-dispersed Poisson (negative binomial) distributions (green line). The two peaks at k-mer frequency 35.9 and 71.8 correspond to single copy (heterozygous) regions and two copy (homozygous) regions, respectively. This spectrum indicates that 63% of the genome are present as single copy and 32% as two copies and that the genome size is 164 Mbp. (PDF 19 kb)

Additional file 3: Distribution of SNV ratio in the *D. coronatus* genome. The numbers of SNV in the paired regions were plotted against the lengths of the regions. Individual circles in a) and b) are the data from CDS and introns of individual genes. The circles in c) are the data from individual intergenic regions. The lines show mean densities of SNV: 3.7% for CDS, 7.1% for intron and 5.6% for the other intergenic regions. (PDF 1749 kb)

Additional file 4: Example of structural variations. A part of paired scaffolds (GBrowse screen capture) is shown. The horizontal green solid bars show the regions that have paired counterparts in the genome. Most of the green bars show a good synteny between the two paired scaffolds, but there are some variations with respect to translocation and inversion. The red circle on the right indicates an inversion, and the red circle on the left shows that the paired sequence corresponding to this green bar is located at another scaffold (translocation). (PDF 24 kb)

Additional file 5: Estimation of nuclear DNA amount by flowcytometry. The histogram of relative DNA content was obtained after flow cytometric analysis of propidium iodide-stained nuclei of a) *C. elegans* and b) *D. coronatus*, c) *D. melanogaster* and d) *D. coronatus* + *D. melanogaster*. *C. elegans* (100 Mbp ×2/nuleus) and *D. melanogaster* (140 Mbp ×2/nuleus) served as reference standard. X-axis: relative nuclear DNA content and Y-axis: number of events. (PDF 111 kb)

Additional file 6: Microscopic measurements of fluorescently labeled nuclear DNA. The fluorescent intensity of ventral nerve cord (VNC) nuclei of *D. coronatus* and sperm and VNC of *C. elegans* stained by Hoechst 33342 were measured as shown in Additional file 9. The y-axis indicates the fluorescent intensity (arbitrary unit: AU) of VNC nuclei in *D. coronatus* (grey circle), sperm and VNC of *C. elegans* (white and black circle, respectively). The amount of *D. coronatus* nuclear DNA was estimated to be 146 Mbp by interpolation of the average fluorescent intensities using *C. elegans* sperm (100 Mbp) and VNC (200 Mbp) as internal standards. (PDF 27 kb)

Additional file 7: Table S3. RNA gene annotations. Genes for rRNA, tRNA and RNA families are listed. **Table S4.** Splice leader sequences found in EST libraries. **Table S5.** List of transposon-like sequences. RepeatModeler identified 754 repeat sequences in the *D. coronatus* genome. The repeat sequences were filtered and classified by REPCLASS. As a result, 423 sequences were retained based on the criteria described in [89] and 104 sequences were classified into 4 categories of transposon-like sequences. (DOCX 35 kb)

Additional file 8: Schematic representation of orthologous gene relationships between *D. coronatus* (Dc) and *C. elegans* (Ce). (2:1): Paired genes (doubleton) in Dc are orthologous to a single gene in Ce. (2:2): Paired genes in Dc is orthologous to a gene family in Ce probably duplicated in the *C. elegans* lineage. (3+:2): Two doubletons in Dc are orthologous to a gene family in Ce. (1:1): A gene without homologous partner (singleton) in Dc is orthologous to a single gene in Ce. (2:0): Paired genes in Dc do not have an ortholog in Ce. (PDF 128 kb)

Additional file 9: Supplementary text and methods. Supplementary description for signal transduction pathways and RNAi pathways, and Supplementary methods for microscopic measurements of fluorescently labeled nuclear DNA are given. (DOCX 32 kb)

Additional file 10: Protein coding gene annotations. **Table S6.** The predicted protein coding genes of *D. coronatus* are listed with their annotations. **Table S7.** The orthologs for *C. elegans* genes are shown. (XLSX 19699 kb)

Additional file 11: Phylogenetic analysis for meiosis-specific genes. Phylogenetic analysis of (1) MSH-2, (2) MSH-4 (HIM-14), (3) MSH-5, (4) MSH-6, (5) RAD-51 and (6) SPO-11 are shown, indicating that orthologs of these *C. elegans* meiotic-specific genes are present in the *D. cornatus* genome mostly in pairs. (PDF 151 kb)

Additional file 12: Histogram of sequence read coverage. The sequence reads from the Miseq library were remapped to the scaffolds by bwa (option: mem, version: 0.7.13-r1126). The mean depth of sequence reads for every 1kbp segments were counted and are shown as a histogram. The distribution of sequence reads is unimodal with the peak at 41.9X coverage. There is no significant peak at twofold higher coverage (84X), indicating that rarely two different regions are assembled together. (PDF 12 kb)

Additional file 13: 3′EST clustering program (cluster3.sh). This shell script originally written for *C.elegans* EST analysis was used. (TXT 5 kb)

Additional file 14: 3D images of DAPI stained chromosomes in oocytes and embryos of *D. coronatus*. 3D images were reconstructed by 3DView software in the FLUOLOVIEW system (Olympus, Tokyo, Japan) from the confocal images obtained for Fig. 2c and f. **Movie S1** and **Movie S2** correspond to Fig. 2c (oocyte) and Fig. 2f (embryo), respectively. (PPTX 437 kb)

## Abbreviations

AU: Arbitrary unit
bp: Base pairs
CDS: Coding sequence
DAPI: 4′,6-diamidino-2-phenylindole dihydrochloride
EST: Expressed sequence tag
FPKM: Fragments per kilo bases of transcript per million fragments sequenced
In/Dels: Insertions and deletions
kbp: Kilo base pairs
Mbp: Mega base pairs
NGS: Next generation sequencing
PI: Propidium iodide
PTH: Permanent translocation heterozygosity
SC: Synaptonemal complex
SL: Spliced leader
SNV: Single nucleotide variations
VNC: Ventral nerve cord
WGD: Whole genome duplication
