# Supplementary material for: Genome analysis of Diploscapter coronatus: insights into molecular peculiarities of a nematode with parthenogenetic reproduction
Source: BMC Genomics. 2017 Jun 24;18:478. doi: 10.1186/s12864-017-3860-x (PMC5483258; doi:10.1186/s12864-017-3860-x)
Supplement: Supplementary file 1 — Sequence reads for analysis. The numbers of genome shotgun reads, RNA-seq reads and ESTs are shown. Table S2. Genome assembly statistics. Information on genome assembly, paired regions between scaffolds, SNVs in the paired regions, In/Dels in the paired regions and BUSCO assessment results are shown. (DOCX 36 kb) [file 12864_2017_3860_MOESM1_ESM.docx]

**Table S1. Sequence reads for analysis**

**Genome shotgun reads**

| Library | Read length (bases) | | | | Read numbers | Insert sizes*  (bases) | |
| --- | --- | --- | --- | --- | --- | --- | --- |
|  | total | minimum | median | maximum |  | mean | std. dev. |
| Sanger shotgun | 783,036,642 | 63 | 691 | 1,230 | 1,211,621 | 2,000 | 1,200 |
| Fosmid ends | 40,381,620 | 64 | 785 | 1,160 | 53,299 | 40,000 | 3,600 |
| 454 | 1,168,522,741 | 64 | 419 | 807 | 2,941,927 |  |  |
| Miseq | 6,810,945,748 | 53 | 249 | 250 | 29,047,682 | 650 | 350 |

*The insert sizes were roughly estimated in order to supply to the assembler as parameters.

**RNA-seq reads**

| Raw reads | Merged sequences | Mapped | Uniquely mapped |
| --- | --- | --- | --- |
| 281,765,048 | 130,699,565 | 118,815,867 | 104,679,506 |

**ESTs**

| Library | | Read lengths (bases) | | | | Read numbers | | |
| --- | --- | --- | --- | --- | --- | --- | --- | --- |
|  |  | mean | minimum | median | maximum | total | cleaned | mapped |
| NDK | 5' | 460 | 101 | 488 | 850 | 38,769 | 38,109 | 36,067 |
|  | 3' | 488 | 100 | 492 | 1,055 | 39,759 | 38,782 | 36,488 |
| NDF | 5' | 626 | 101 | 654 | 995 | 33,940 | 33,792 | 33,179 |
|  | 3' | 702 | 100 | 747 | 1,119 | 37,173 | 36,628 | 35,970 |
| NDV | 5' | 614 | 101 | 665 | 1,140 | 129,837 | 129,044 | 128,840 |
|  | 3' | 641 | 100 | 690 | 1,200 | 129,418 | 128,576 | 128,382 |
| total |  | 605 | 100 | 654 | 1,200 | 408,896 | 404,931 | 398,926 |

**Table S2. Genome assembly statistics**

**Genome assembly**

|  | Size (bp) | | | | | Number |
| --- | --- | --- | --- | --- | --- | --- |
|  | total | minimum | median | maximum | N50 |  |
| Scaffold | 170,470,384 | 2,026 | 63,175 | 3,561,896 | 1,007,652 | 511 |
| Contig | 169,424,175 | 849 | 79,864 | 1,740,259 | 487,148 | 867 |
| Gap | 1,046,209 | 20* | 20 | 34,749 | 14,841 | 356 |

*The minimum gap size was set by the assembler.

**Paired regions between scaffolds**

| Number | Length (bp) | | | |
| --- | --- | --- | --- | --- |
|  | total | minimum | median | maximum |
| 6,690 | 152,151,424 | 1,035 | 9,729 | 250,158 |

**SNVs in the paired regions**

|  | Count |
| --- | --- |
| Transition | 6,289,356 |
| Transversion | 2,393,802 |
| Ambiguous* | 2,815 |
| total | 8,685,973 |

* Mismatches involving the ambiguous nucleotide "N".

**In/Dels in the paired regions**

|  | Counts | Lengths (bp) | | | |
| --- | --- | --- | --- | --- | --- |
|  |  | mean | minimum | median | maximum |
| Insertions | 498,971 | 6.73 | 1 | 2 | 991 |
| Deletions | 498,372 | 6.71 | 1 | 2 | 991 |
| total | 997,343 |  |  |  |  |

**BUSCO assessment results**

|  | C | D | F | M | n |
| --- | --- | --- | --- | --- | --- |
| *D. coronatus* | 83% | 80% | 3.2% | 13% | 843 |
| *C. elegans** | 90% | 11% | 1.7% | 7.5%, | 843 |

(C: complete, D: duplicated, F: fragmented, M: missing, n: gene number).

* [28]

The higher value in column D is thought to reflect the paired structure.
