# Supplementary figures and images for: Genome analysis of Diploscapter coronatus: insights into molecular peculiarities of a nematode with parthenogenetic reproduction
Source: BMC Genomics. 2017 Jun 24;18:478. doi: 10.1186/s12864-017-3860-x (PMC5483258; doi:10.1186/s12864-017-3860-x)

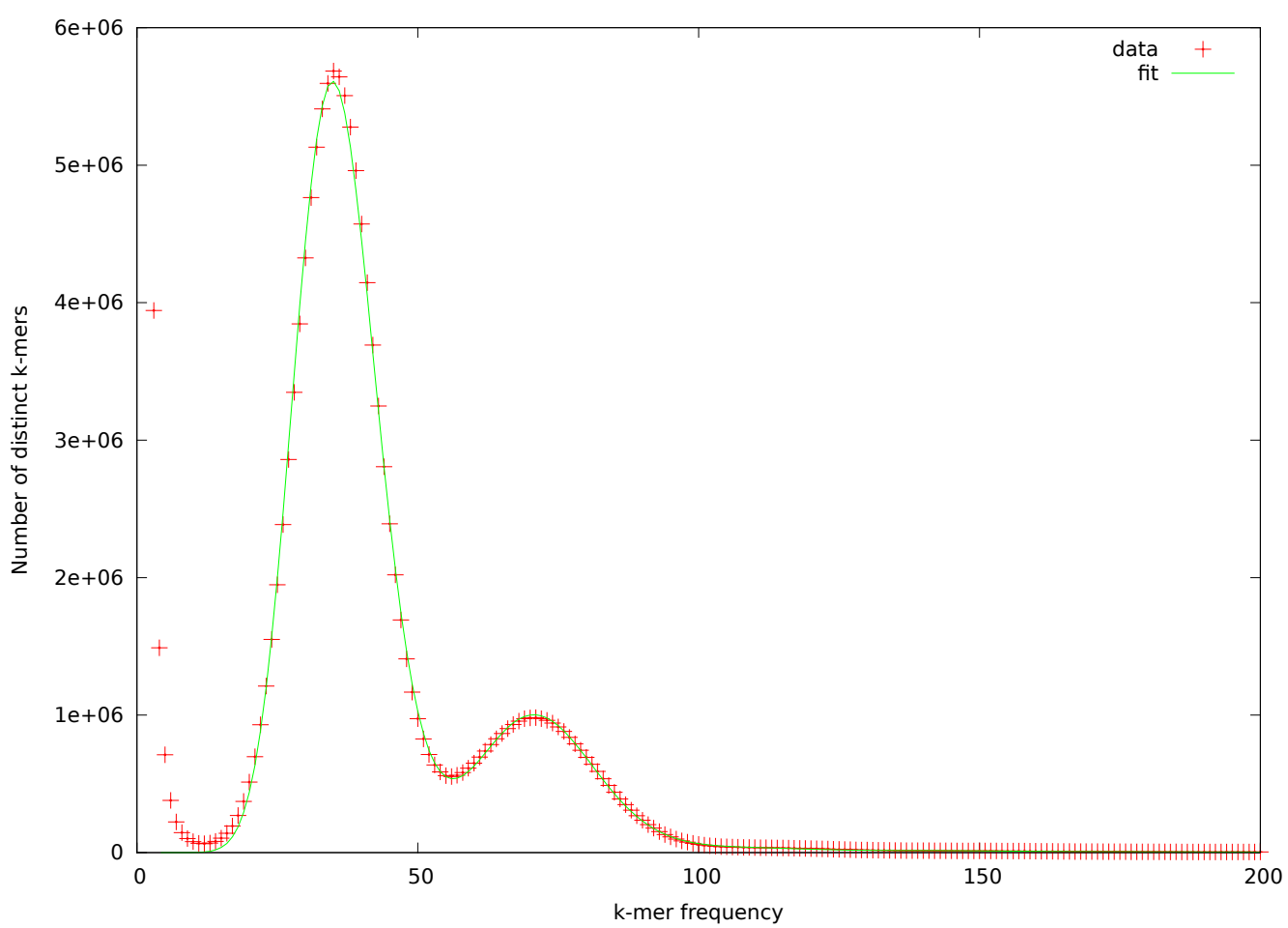

Supplement: Supplementary file 2 — k-mer distribution analysis. k-mer distribution in the Miseq library was analyzed with kmerspectrumanalyzer (version b584039 with jellyfish version 2.0.0, numpy version 1.8.1 and scipy version 0.12.0) [114]. The frequency of each 21-mer in the library was measured and the number of distinct 21-mers for each frequency is plotted (red cross). This frequency spectrum was fitted as a mixture of over-dispersed Poisson (negative binomial) distributions (green line). The two peaks at k-mer frequency 35.9 and 71.8 correspond to single copy (heterozygous) regions and two copy (homozygous) regions, respectively. This spectrum indicates that 63% of the genome are present as single copy and 32% as two copies and that the genome size is 164 Mbp. (PDF 19 kb) [file 12864_2017_3860_MOESM2_ESM.pdf]

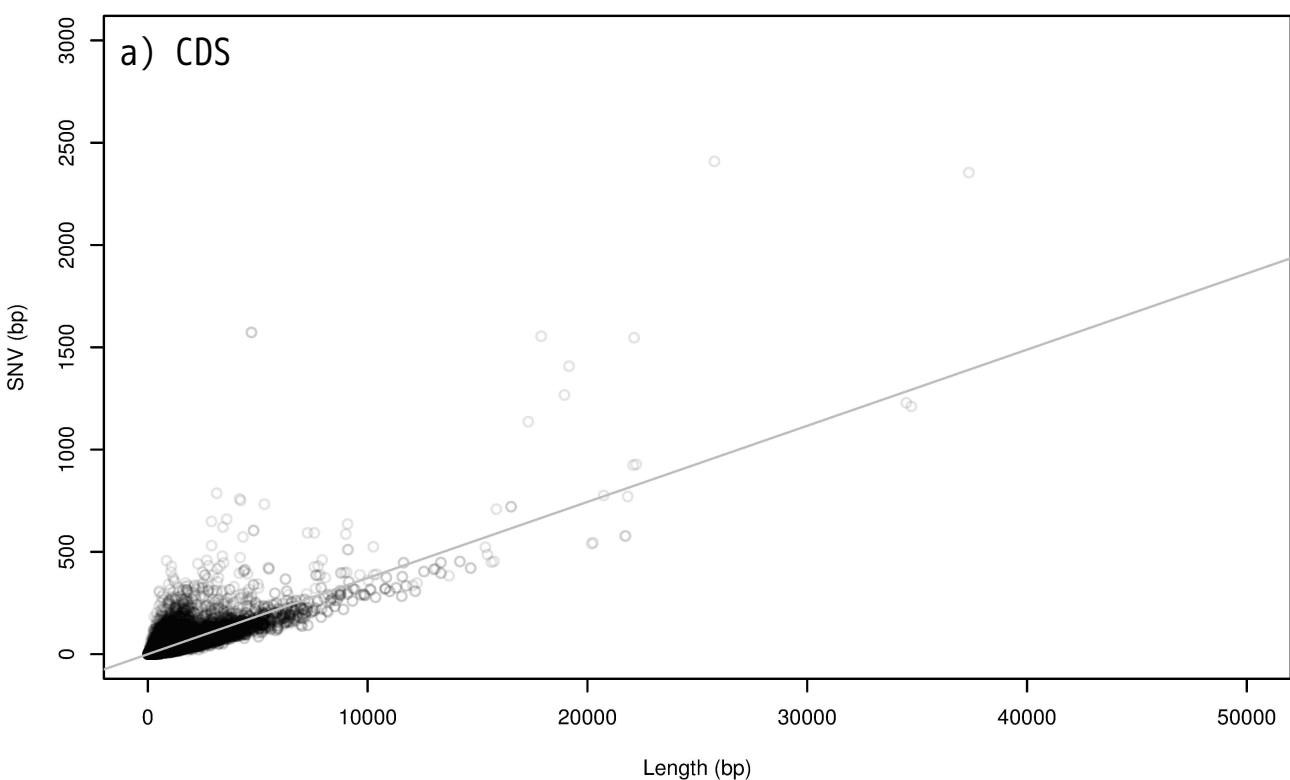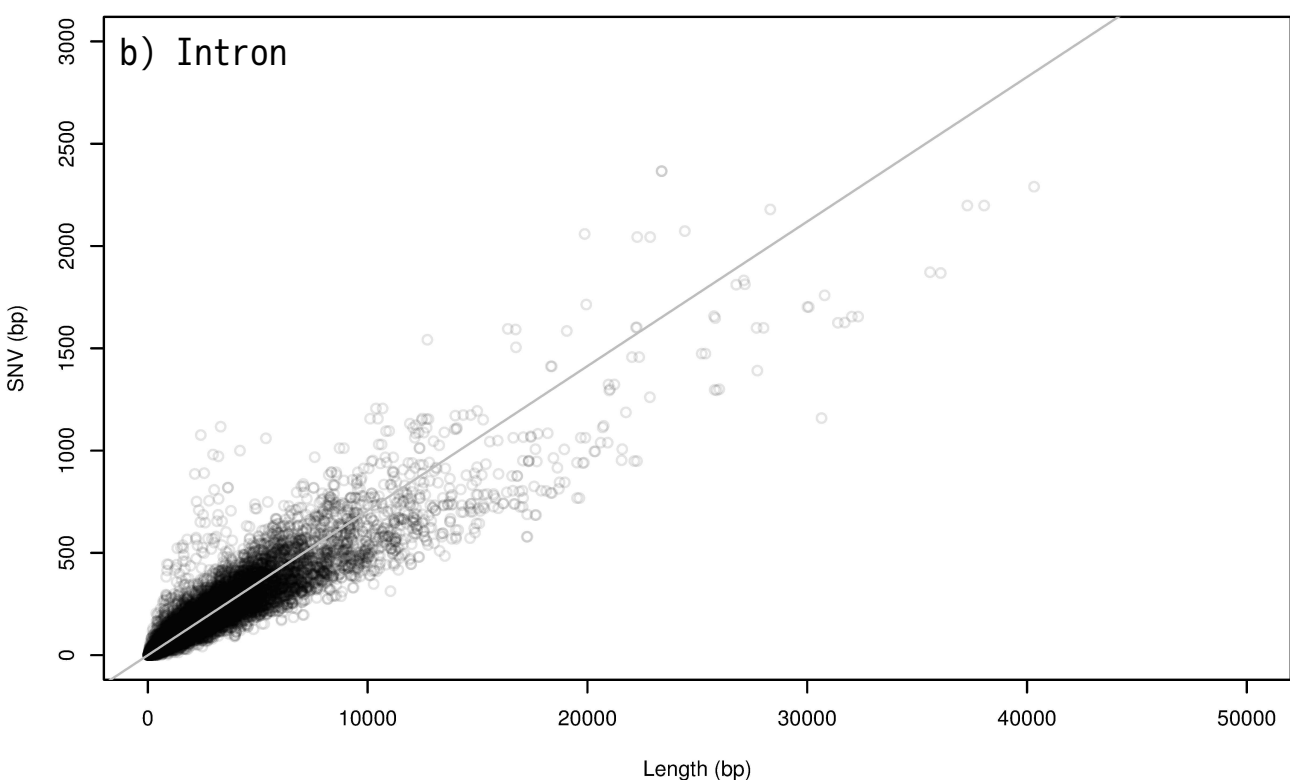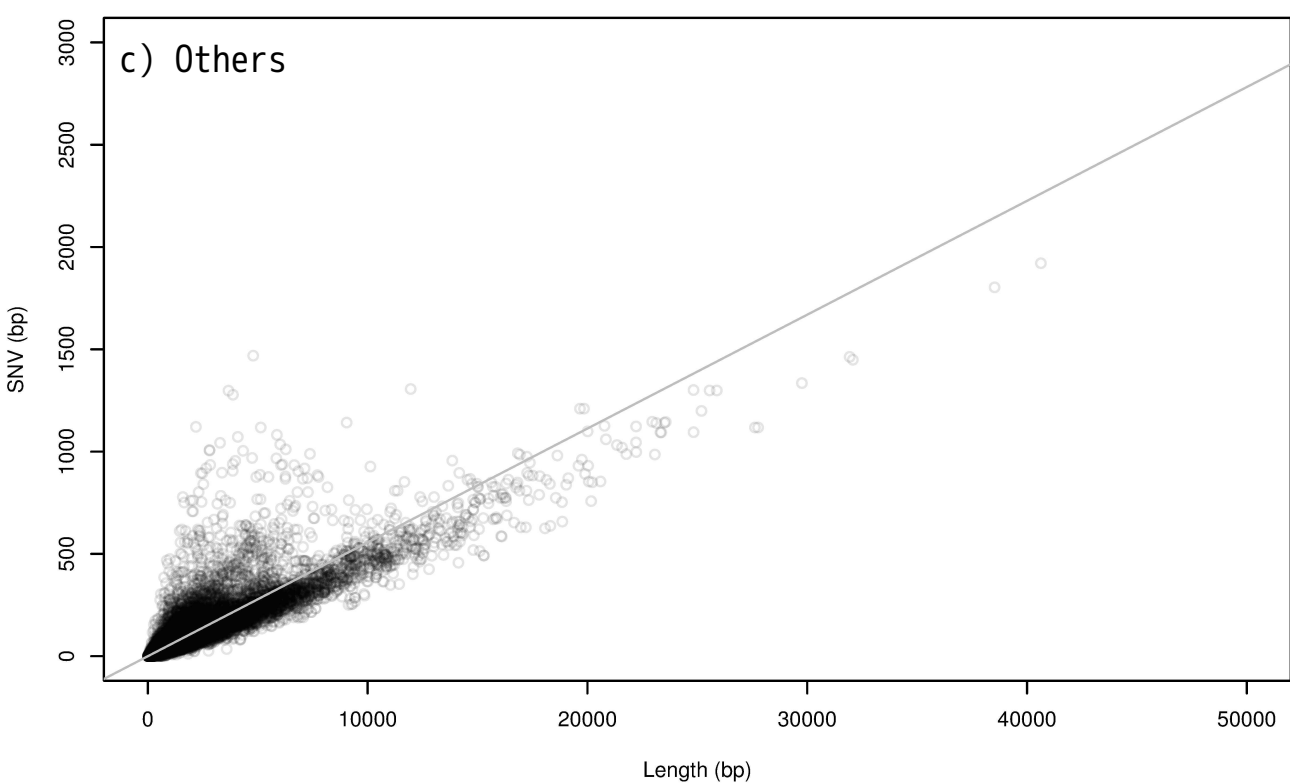

Supplement: Supplementary file 3 — Distribution of SNV ratio in the D. coronatus genome. The numbers of SNV in the paired regions were plotted against the lengths of the regions. Individual circles in a) and b) are the data from CDS and introns of individual genes. The circles in c) are the data from individual intergenic regions. The lines show mean densities of SNV: 3.7% for CDS, 7.1% for intron and 5.6% for the other intergenic regions. (PDF 1749 kb) [file 12864_2017_3860_MOESM3_ESM.pdf]

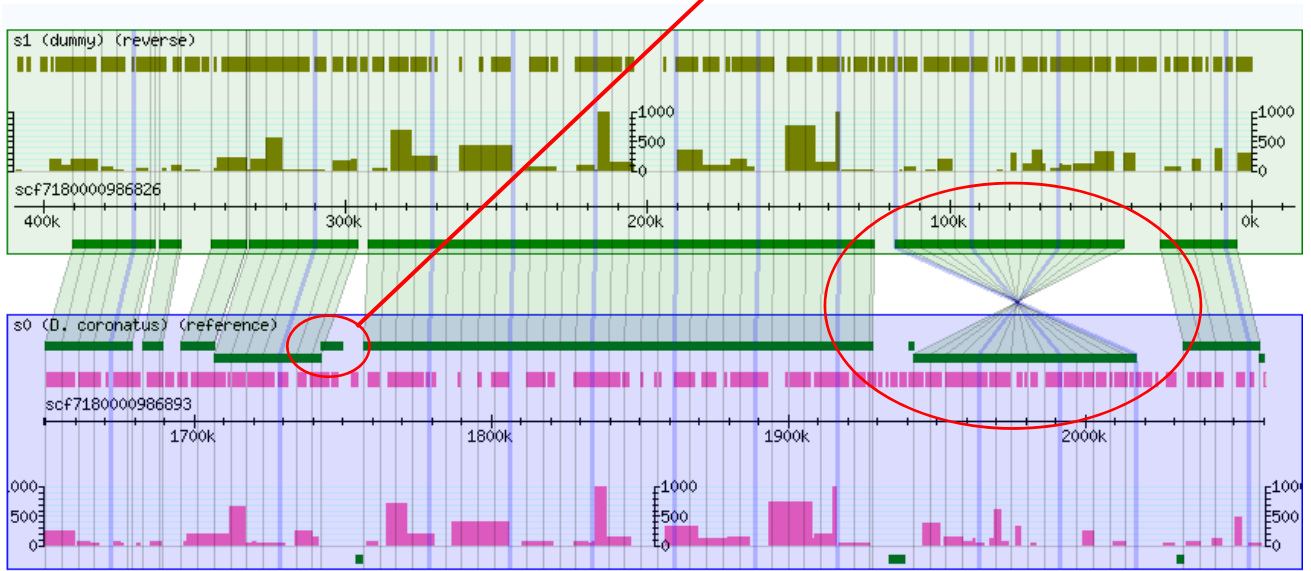

Supplement: Supplementary file 4 — Example of structural variations. A part of paired scaffolds (GBrowse screen capture) is shown. The horizontal green solid bars show the regions that have paired counterparts in the genome. Most of the green bars show a good synteny between the two paired scaffolds, but there are some variations with respect to translocation and inversion. The red circle on the right indicates an inversion, and the red circle on the left shows that the paired sequence corresponding to this green bar is located at another scaffold (translocation). (PDF 24 kb) [file 12864_2017_3860_MOESM4_ESM.pdf]

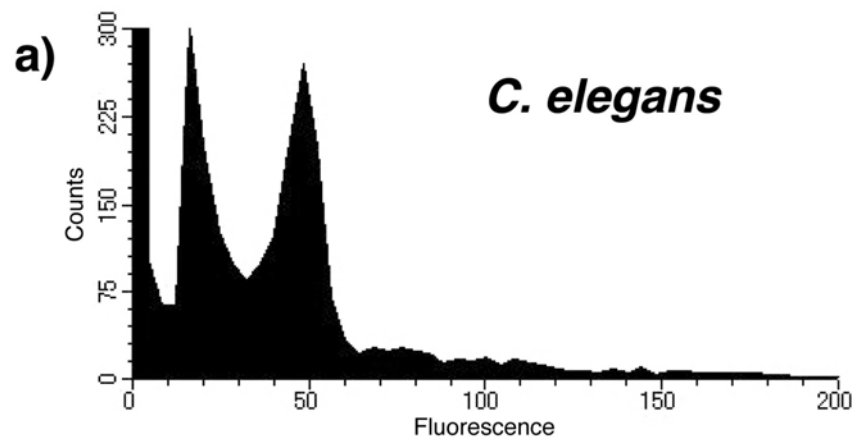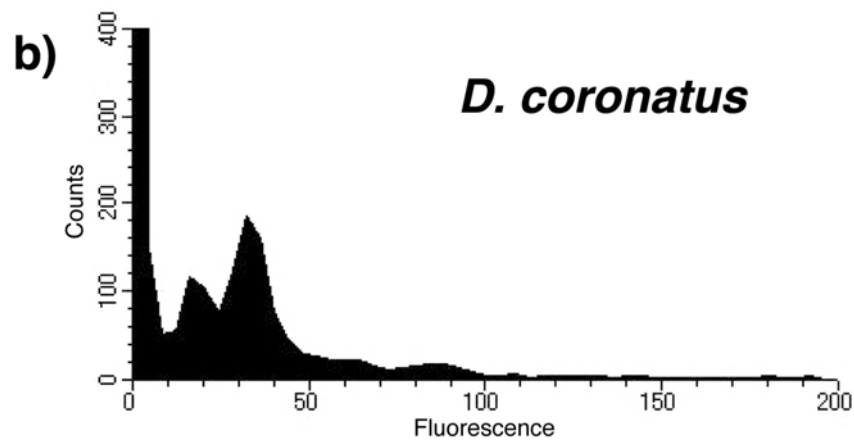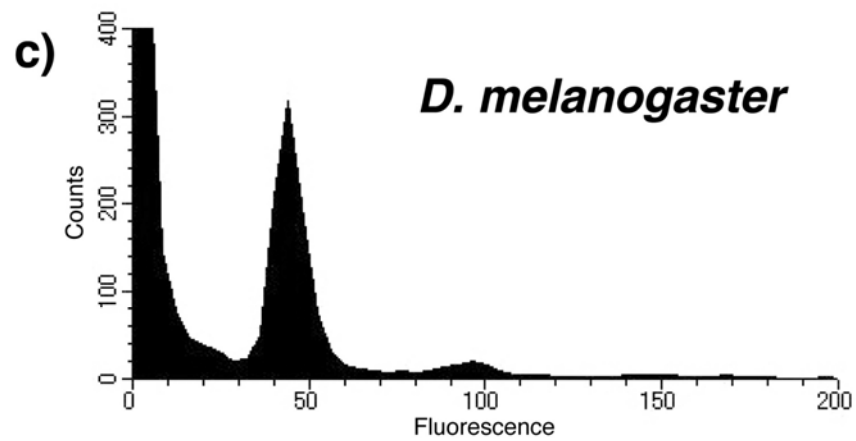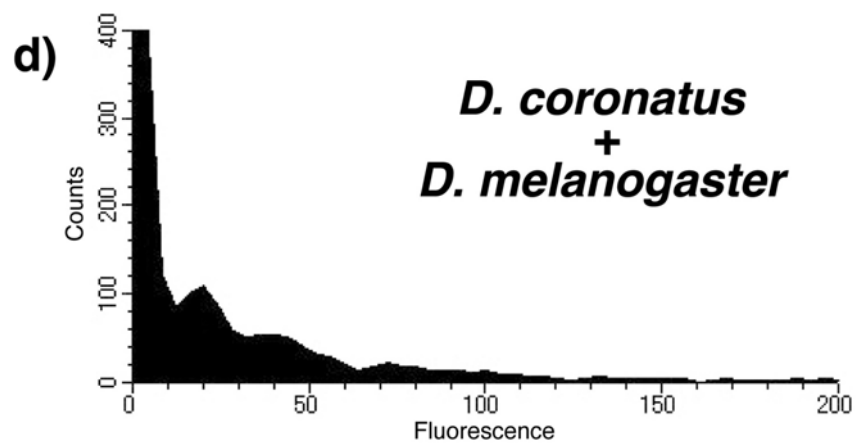

Supplement: Supplementary file 5 — Estimation of nuclear DNA amount by flowcytometry. The histogram of relative DNA content was obtained after flow cytometric analysis of propidium iodide-stained nuclei of a) C. elegans and b) D. coronatus, c) D. melanogaster and d) D. coronatus + D. melanogaster. C. elegans (100 Mbp ×2/nuleus) and D. melanogaster (140 Mbp ×2/nuleus) served as reference standard. X-axis: relative nuclear DNA content and Y-axis: number of events. (PDF 111 kb) [file 12864_2017_3860_MOESM5_ESM.pdf]

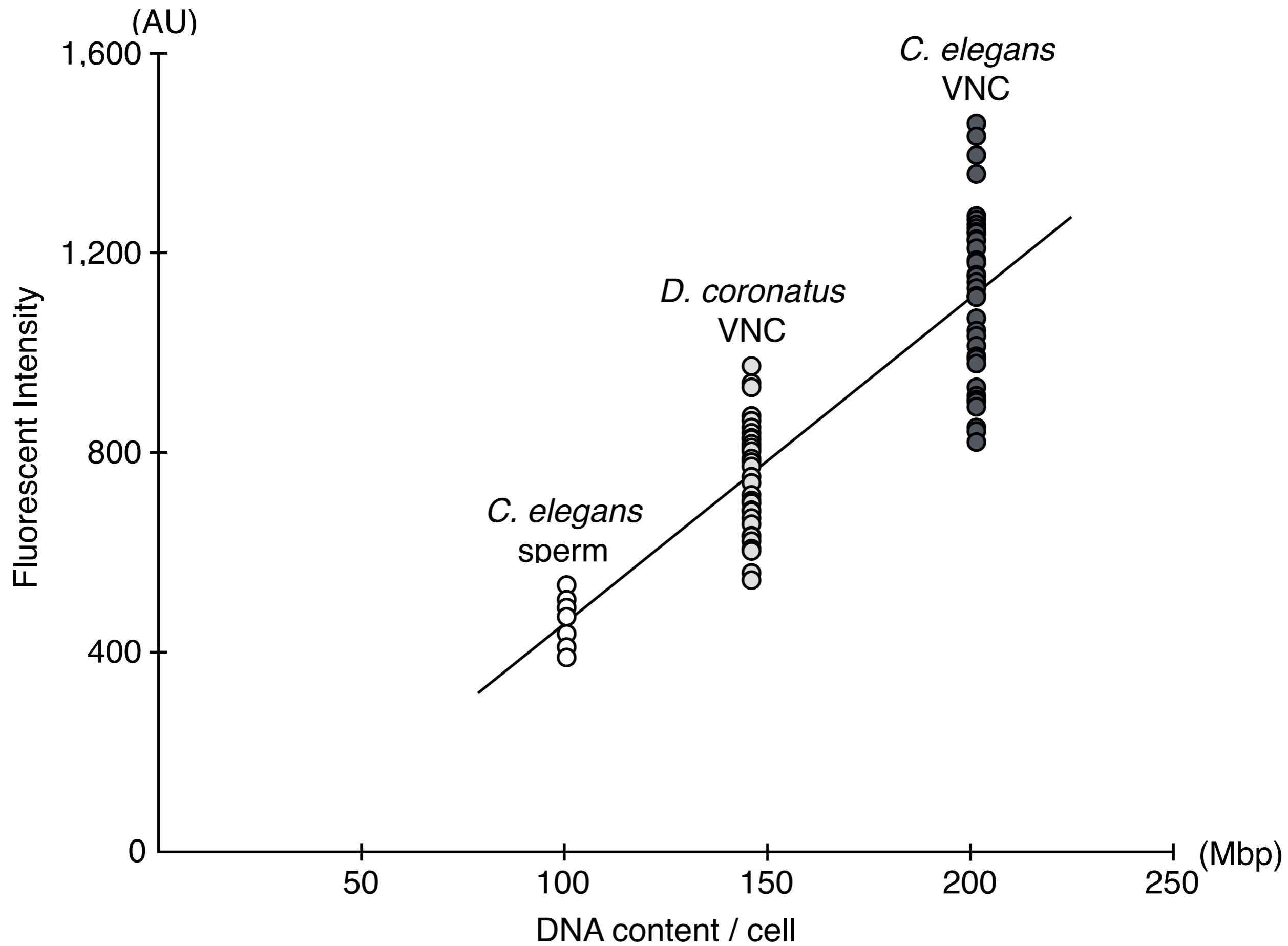

Supplement: Supplementary file 6 — Microscopic measurements of fluorescently labeled nuclear DNA. The fluorescent intensity of ventral nerve cord (VNC) nuclei of D. coronatus and sperm and VNC of C. elegans stained by Hoechst 33342 were measured as shown in Additional file 9. The y-axis indicates the fluorescent intensity (arbitrary unit: AU) of VNC nuclei in D. coronatus (grey circle), sperm and VNC of C. elegans (white and black circle, respectively). The amount of D. coronatus nuclear DNA was estimated to be 146 Mbp by interpolation of the average fluorescent intensities using C. elegans sperm (100 Mbp) and VNC (200 Mbp) as internal standards. (PDF 27 kb) [file 12864_2017_3860_MOESM6_ESM.pdf]

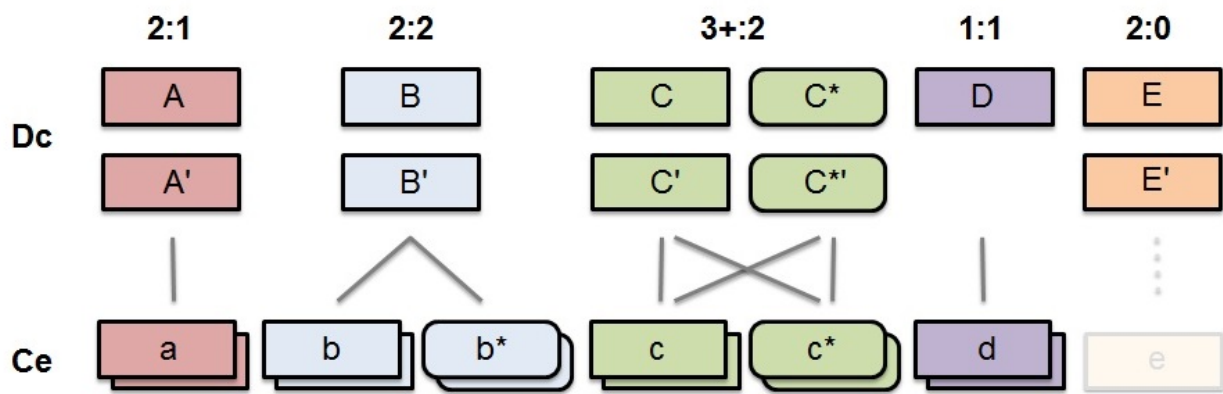

Supplement: Supplementary file 8 — Schematic representation of orthologous gene relationships between D. coronatus (Dc) and C. elegans (Ce). (2:1): Paired genes (doubleton) in Dc are orthologous to a single gene in Ce. (2:2): Paired genes in Dc is orthologous to a gene family in Ce probably duplicated in the C. elegans lineage. (3+:2): Two doubletons in Dc are orthologous to a gene family in Ce. (1:1): A gene without homologous partner (singleton) in Dc is orthologous to a single gene in Ce. (2:0): Paired genes in Dc do not have an ortholog in Ce. (PDF 128 kb) [file 12864_2017_3860_MOESM8_ESM.pdf]

# (1) MSH-2

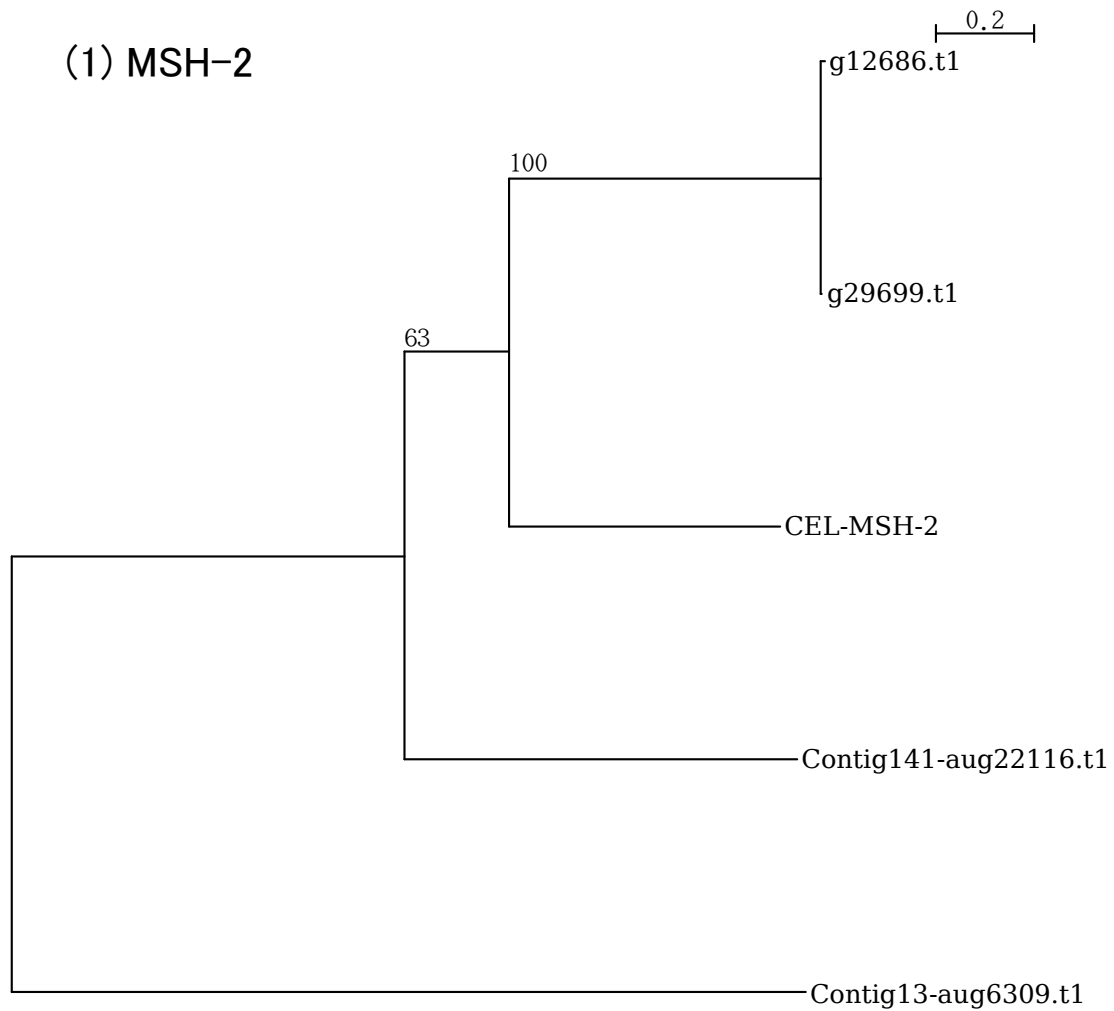

(2) MSH-5 (HIM-14)

0.1

Ppa-HIM-14

CEL-HIM-14

g20705.t1

100

g27186.t1

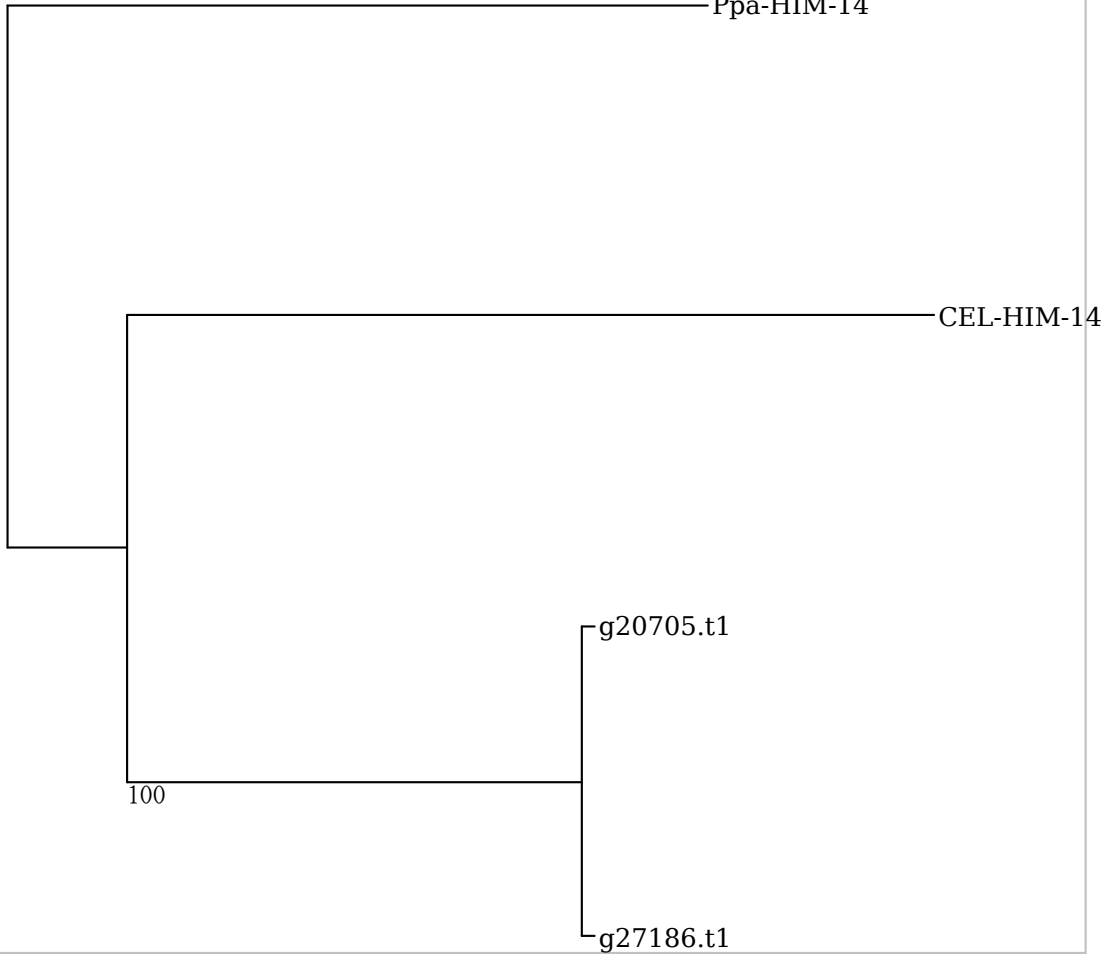

### (3) MSH-5

0.1

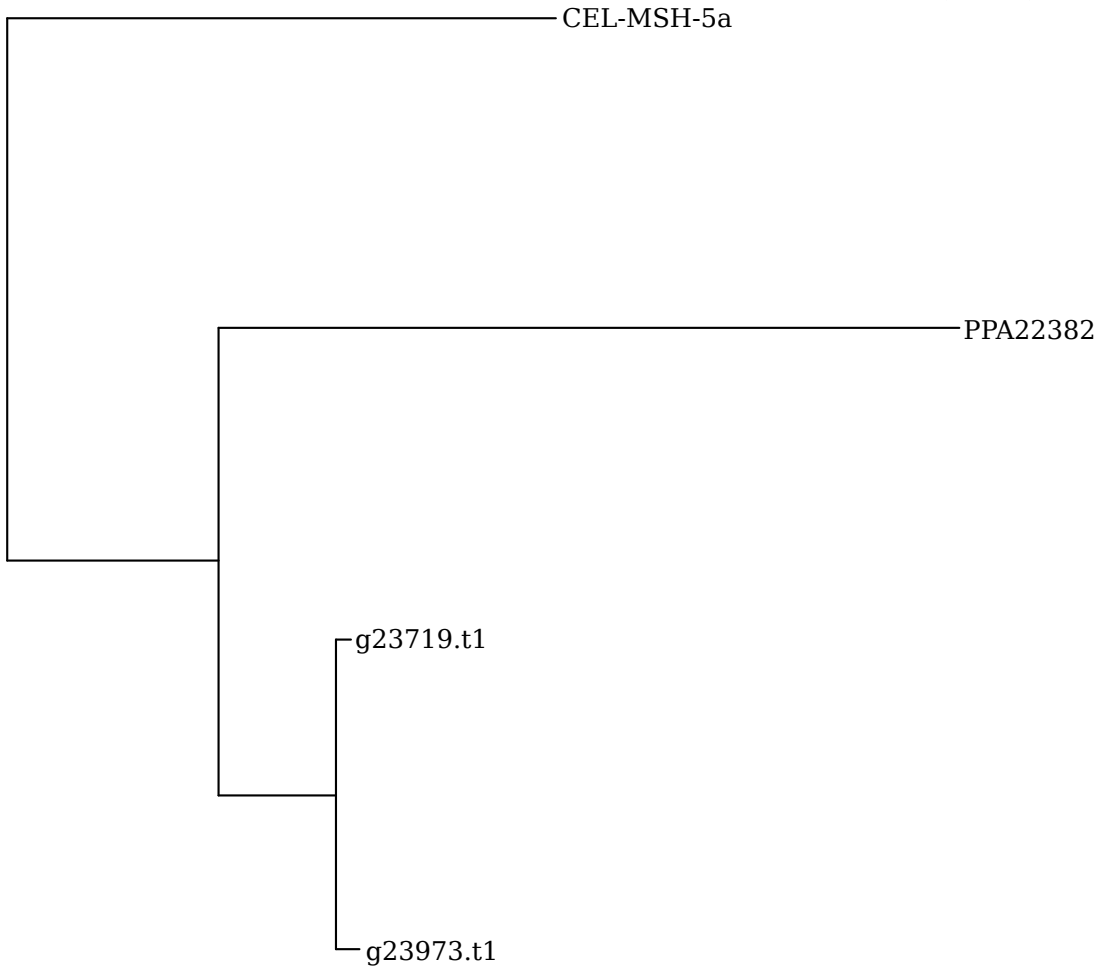

#### (4) MSH-6

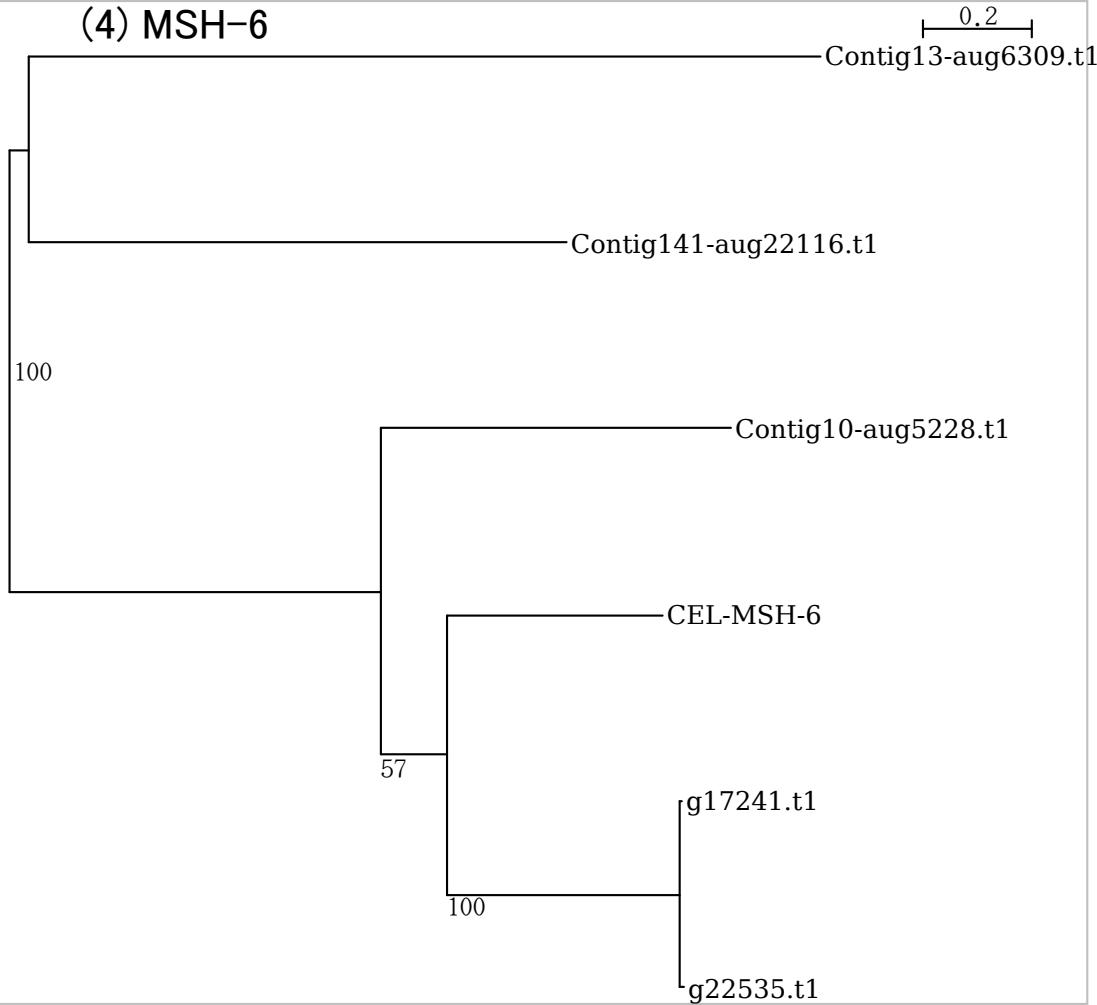

# (5) RAD-51

0.1

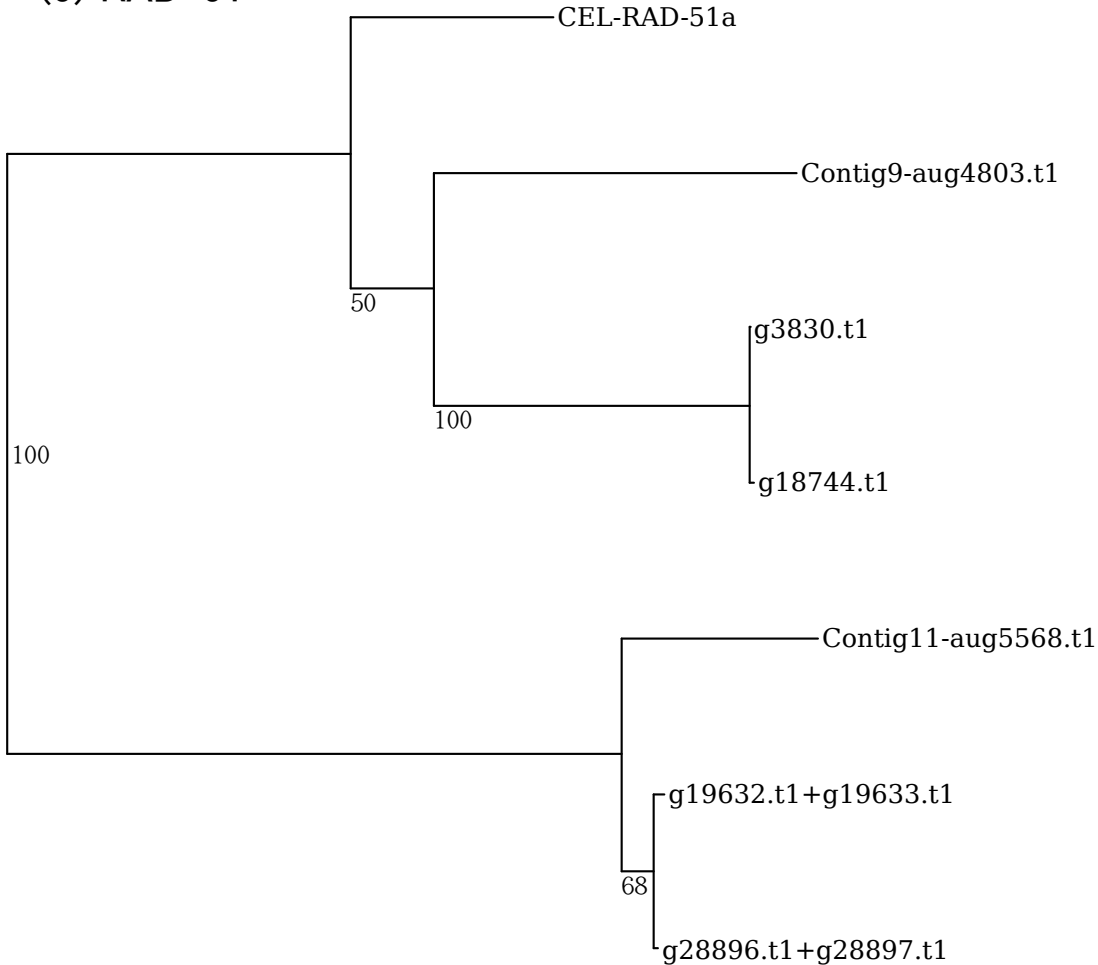

# (6) SPO-11

0.1

CEL-SPO-11a

Ppa-SPO-11

g26124.t1

97

g18275.t1

4

g26123.t1

34

g18276.t1

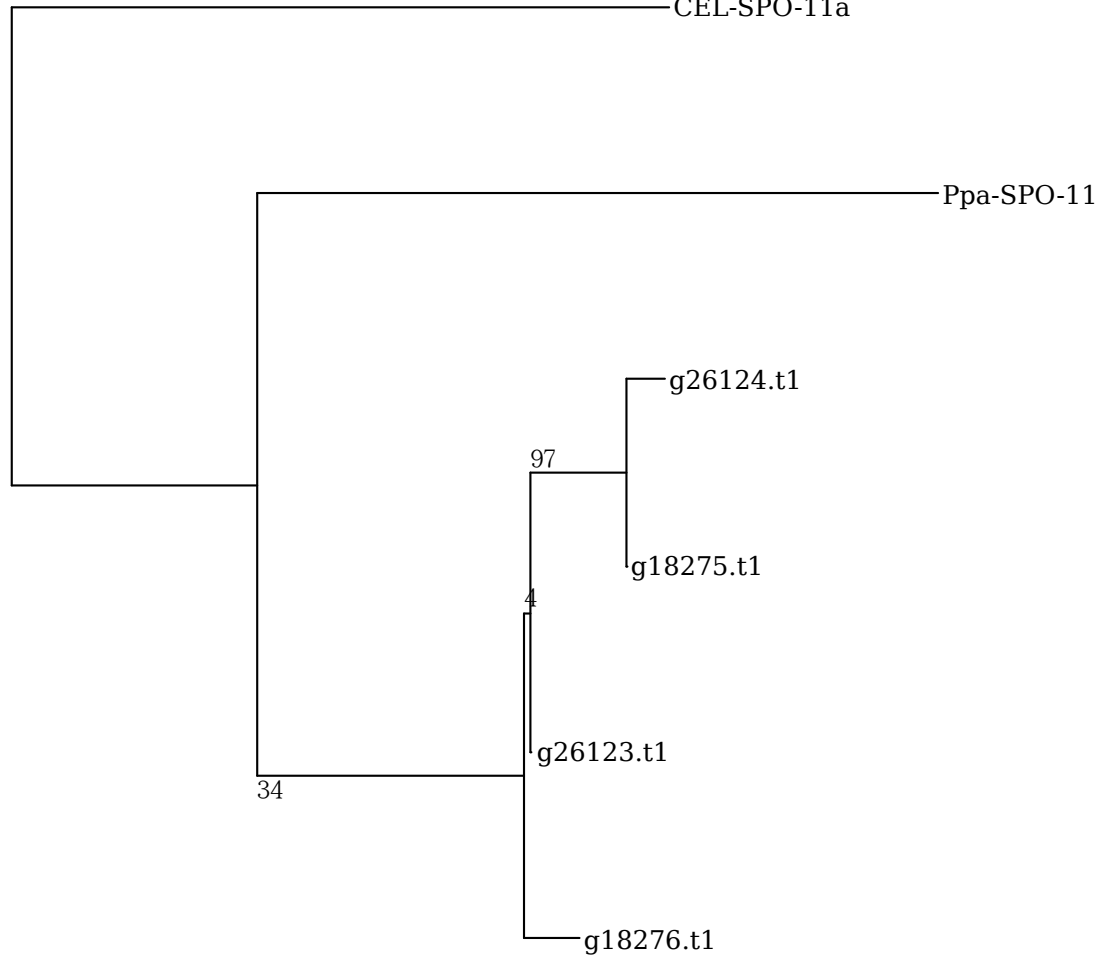

Supplement: Supplementary file 11 — Phylogenetic analysis for meiosis-specific genes. Phylogenetic analysis of (1) MSH-2, (2) MSH-4 (HIM-14), (3) MSH-5, (4) MSH-6, (5) RAD-51 and (6) SPO-11 are shown, indicating that orthologs of these C. elegans meiotic-specific genes are present in the D. cornatus genome mostly in pairs. (PDF 151 kb) [file 12864_2017_3860_MOESM11_ESM.pdf]

Histogram of sequence read coverage

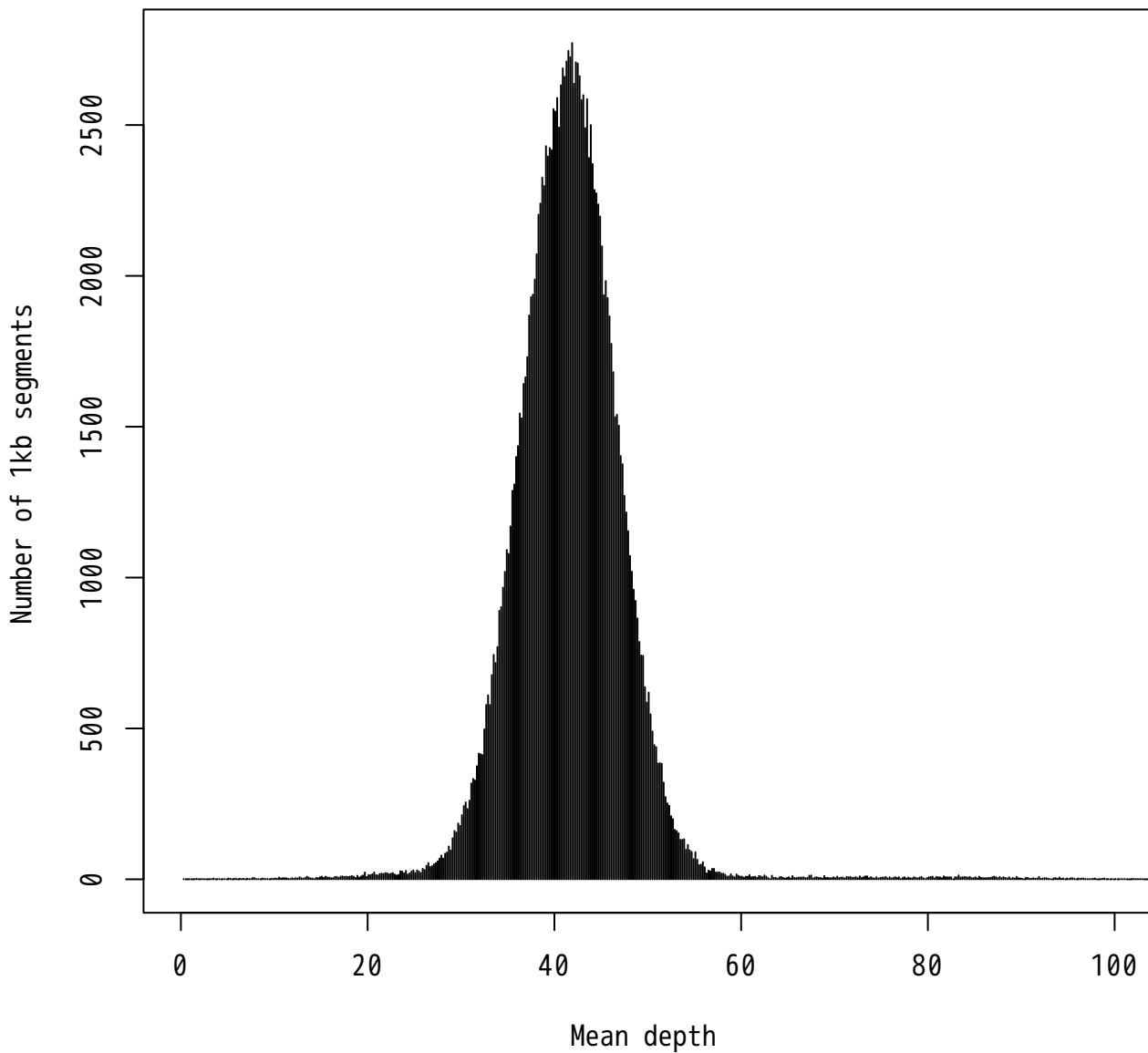

Supplement: Supplementary file 12 — Histogram of sequence read coverage. The sequence reads from the Miseq library were remapped to the scaffolds by bwa (option: mem, version: 0.7.13-r1126). The mean depth of sequence reads for every 1kbp segments were counted and are shown as a histogram. The distribution of sequence reads is unimodal with the peak at 41.9X coverage. There is no significant peak at twofold higher coverage (84X), indicating that rarely two different regions are assembled together. (PDF 12 kb) [file 12864_2017_3860_MOESM12_ESM.pdf]
