# Supplementary material for: Genome analysis of Diploscapter coronatus: insights into molecular peculiarities of a nematode with parthenogenetic reproduction
Source: BMC Genomics. 2017 Jun 24;18:478. doi: 10.1186/s12864-017-3860-x (PMC5483258; doi:10.1186/s12864-017-3860-x)
Supplement: Supplementary file 7 — RNA gene annotations. Genes for rRNA, tRNA and RNA families are listed. Table S4. Splice leader sequences found in EST libraries. Table S5. List of transposon-like sequences. RepeatModeler identified 754 repeat sequences in the D. coronatus genome. The repeat sequences were filtered and classified by REPCLASS. As a result, 423 sequences were retained based on the criteria described in [89] and 104 sequences were classified into 4 categories of transposon-like sequences. (DOCX 35 kb) [file 12864_2017_3860_MOESM7_ESM.docx]

**Table S3: RNA gene annotation**

**rRNA gene**

| Name | Counts |
| --- | --- |
| 18s_rRNA | 5 |
| 28s_rRNA | 6 |
| 8s_rRNA* | 67 |

*In the output of RNAmmer, eukaryotic 5S RNA is named as 8s_RNA.

**tRNA genes**

| Type | Anticodon | Counts |
| --- | --- | --- |
| Ala | AGC | 7 |
| Ala | CGC | 4 |
| Ala | TGC | 2 |
| Arg | ACG | 4 |
| Arg | CCG | 4 |
| Arg | CCT | 2 |
| Arg | TCG | 4 |
| Arg | TCT | 4 |
| Asn | ATT | 1 |
| Asn | GTT | 10 |
| Asp | GTC | 9 |
| Cys | GCA | 2 |
| Gln | CTG | 8 |
| Gln | TTG | 2 |
| Glu | CTC | 8 |
| Glu | TTC | 8 |
| Gly | CCC | 4 |
| Gly | GCC | 8 |
| Gly | TCC | 10 |
| His | GTG | 6 |
| Ile | AAT | 7 |
| Ile | GAT | 2 |
| Ile | TAT | 2 |
| Leu | AAG | 6 |
| Leu | CAA | 5 |
| Leu | CAG | 5 |
| Leu | TAA | 5 |
| Lys | CTT | 10 |
| Lys | TTT | 7 |
| Met | CAT | 19 |
| Phe | GAA | 6 |
| Pro | AGG | 6 |
| Pro | CGG | 3 |
| Pro | TGG | 10 |
| Ser | AGA | 6 |
| Ser | CGA | 5 |
| Ser | GCT | 5 |
| Ser | TGA | 2 |
| Thr | AGT | 11 |
| Thr | CGT | 10 |
| Thr | TGT | 5 |
| Trp | CCA | 4 |
| Tyr | GTA | 7 |
| Val | AAC | 4 |
| Val | CAC | 4 |
| Val | TAC | 2 |
| Pseudo | CGG | 1 |
| Pseudo | TAA | 1 |
| Pseudo | TAG | 1 |
| Pseudo | TAT | 2 |
| Pseudo | TGA | 1 |
| Pseudo | TGT | 2 |

**Top 75 RNA families**

| Name | Counts |
| --- | --- |
| tRNA | 239 |
| SL1 | 185 |
| 5S_rRNA | 68 |
| U5 | 51 |
| U2 | 39 |
| U1 | 38 |
| ceN72-3_ceN74-2 | 38 |
| MIR1023 | 37 |
| MIR821 | 37 |
| MIR408 | 36 |
| Histone3 | 31 |
| SL2 | 27 |
| MIR2118 | 25 |
| U4 | 22 |
| mir-42 | 21 |
| MIR530 | 17 |
| SCARNA7 | 17 |
| mir-548 | 15 |
| MIR1027 | 14 |
| K_chan_RES | 12 |
| U3 | 12 |
| tRNA-Sec | 12 |
| ACEA_U3 | 11 |
| MIR811 | 10 |
| U6 | 9 |
| ceN30 | 9 |
| mir-202 | 9 |
| mir-172 | 8 |
| mir-281 | 7 |
| mir-605 | 7 |
| mir-609 | 7 |
| MIR398 | 6 |
| MIR807 | 6 |
| PK-G12rRNA | 6 |
| SSU_rRNA_archaea | 6 |
| SSU_rRNA_bacteria | 6 |
| SSU_rRNA_eukarya | 6 |
| mir-153 | 6 |
| mir-1803 | 6 |
| mir-186 | 6 |
| mir-232 | 6 |
| mir-3179 | 6 |
| mir-48 | 6 |
| mir-576 | 6 |
| mir-81 | 6 |
| ceN28 | 5 |
| mir-32 | 5 |
| mir-449 | 5 |
| MIR1444 | 4 |
| MIR396 | 4 |
| MIR397 | 4 |
| SNORA73 | 4 |
| SNORD14 | 4 |
| SNORD15 | 4 |
| ceN108 | 4 |
| ceN109 | 4 |
| ceN70 | 4 |
| mir-1 | 4 |
| mir-250 | 4 |
| mir-788 | 4 |
| sn3071 | 4 |
| 5_8S_rRNA | 3 |
| RNase_MRP | 3 |
| ceN89 | 3 |
| mir-197 | 3 |
| mir-231 | 3 |
| mir-234 | 3 |
| mir-255 | 3 |
| mir-280 | 3 |
| mir-367 | 3 |
| mir-395 | 3 |
| mir-399 | 3 |
| mir-85 | 3 |
| mir-942 | 3 |
| mir-944 | 3 |

**Table S4: Splice leader sequences found in EST libraries**

| Ce_SL1 | GGTTTAATTACCCAAGTTTGAG |
| --- | --- |
| Ce_SL2 | GGTTTTAACCCAGTTACTCAAG |
| Dc_SL2* | GGTTATTACCCAGTTACTCAAG |

*new sequence

**Table S5: List of transposon-like sequences**

|  | *D. coronatus* | | *C. elegans** | |
| --- | --- | --- | --- | --- |
|  | Number | Average length (bp) | Number | Average length (bp) |
| DNA transposons | 84 | 382 | 107 | 649 |
| Helitron | 1 | 1743 | 5 | 891 |
| LTR | 5 | 256 | 11 | 1403 |
| Non-LTR | 14 | 683 | 23 | 707 |

* Data from *C. elegans* genome [89] for comparison.
