# Supplementary material for: Genome analysis of Diploscapter coronatus: insights into molecular peculiarities of a nematode with parthenogenetic reproduction
Source: BMC Genomics. 2017 Jun 24;18:478. doi: 10.1186/s12864-017-3860-x (PMC5483258; doi:10.1186/s12864-017-3860-x)
Supplement: Supplementary file 9 — Supplementary text and methods. Supplementary description for signal transduction pathways and RNAi pathways, and Supplementary methods for microscopic measurements of fluorescently labeled nuclear DNA are given. (DOCX 32 kb) [file 12864_2017_3860_MOESM9_ESM.docx]

**Supplementary description**

**Peculiarities in Gene repertoire of parthenogenetic nematode**

*Signal transduction pathways*

The major signaling systems are conserved to a remarkable extent in all animals. The *D. coronatus* genome analysis revealed that these have clear orthologs to most of the *C. elegans* genes involved in well-known signaling pathways, i.e. Wnt, TGF-β, IIS (insulin/insulin-like growth factor signaling), heteromeric G proteins, and RTK/Ras/MAPK (Receptor Tyrosine kinase/Ras GTPase/MAP kinase) (Additional file 11) [S1-S5]. We identified two pairs of Notch receptor homologs in the *D. coronatus* genome by Pfam analysis. *C. elegans* has 10 Delta ligand homologs (LAG-2, APX-1, ARG-1 and DSL-1~7) and *D. coronatus* has three pairs of homologs of Delta-related genes identified by Pfam analysis.

*RNAi pathways*

RNAi (RNA interference) is found in many phyla. It controls diverse processes, such as development, metabolism, cell fate, transposon silencing and immunity to viral infection. In *D. coronatus,* we found essentially all RNAi pathway components (Additional file 11). However, our attempts to achieve gene silencing via RNAi have not been successful to date.

**Supplementary methods**

**Microscopic measurements of fluorescently labeled nuclear DNA.**

Adult worms (*D. coronatus* and *C. elegans*) were collected after incubation in distilled water for 2 hours to digest the remaining food bacteria. The worms were mixed and mounted on a poly-Lysine coated glass slide with 5µl of M9 buffer containing 100 µg/ml Hoechst 33342. The sample slides were subsequently placed on dry-ice to freeze and incubated for 10 min at room temperature. The samples were viewed under the Zeiss epifluorescence microscope (Axioplan2). Images of ventral cord nuclei (VNC of *D. coronatus* and *C. elegans*) and sperms (of *C. elegans*) were collected using the ORCA-ER video camera (Hamamatsu Photonics), and analyzed using AQUACOSMOS software (Hamamatsu Photonics).

Reference

S1. Bastiani C, Mendel J: Heterotrimeric G proteins in *C. elegans*. WormBook 2006:1-25.

S2. Gumienny TL, Savage-Dunn C: TGF-beta signaling in *C. elegans*. WormBook 2013:1-34.

S3. Murphy CT, Hu PJ: Insulin/insulin-like growth factor signaling in *C. elegans*. WormBook 2013:1-43.

S4. Sawa H, Korswagen HC: Wnt signaling in *C. elegans*. WormBook 2013:1-30.

S5. Sundaram MV: Canonical RTK-Ras-ERK signaling and related alternative pathways. WormBook 2013:1-38.
