# Supplementary material for: Genome analysis of Diploscapter coronatus: insights into molecular peculiarities of a nematode with parthenogenetic reproduction
Source: BMC Genomics. 2017 Jun 24;18:478. doi: 10.1186/s12864-017-3860-x (PMC5483258; doi:10.1186/s12864-017-3860-x)
Supplement: Supplementary file 14 — 3D images of DAPI stained chromosomes in oocytes and embryos of D. coronatus. 3D images were reconstructed by 3DView software in the FLUOLOVIEW system (Olympus, Tokyo, Japan) from the confocal images obtained for Fig. 2c and f. Movie S1 and Movie S2 correspond to Fig. 2c (oocyte) and Fig. 2f (embryo), respectively. (PPTX 437 kb) [file 12864_2017_3860_MOESM14_ESM.pptx]

## Slide 1
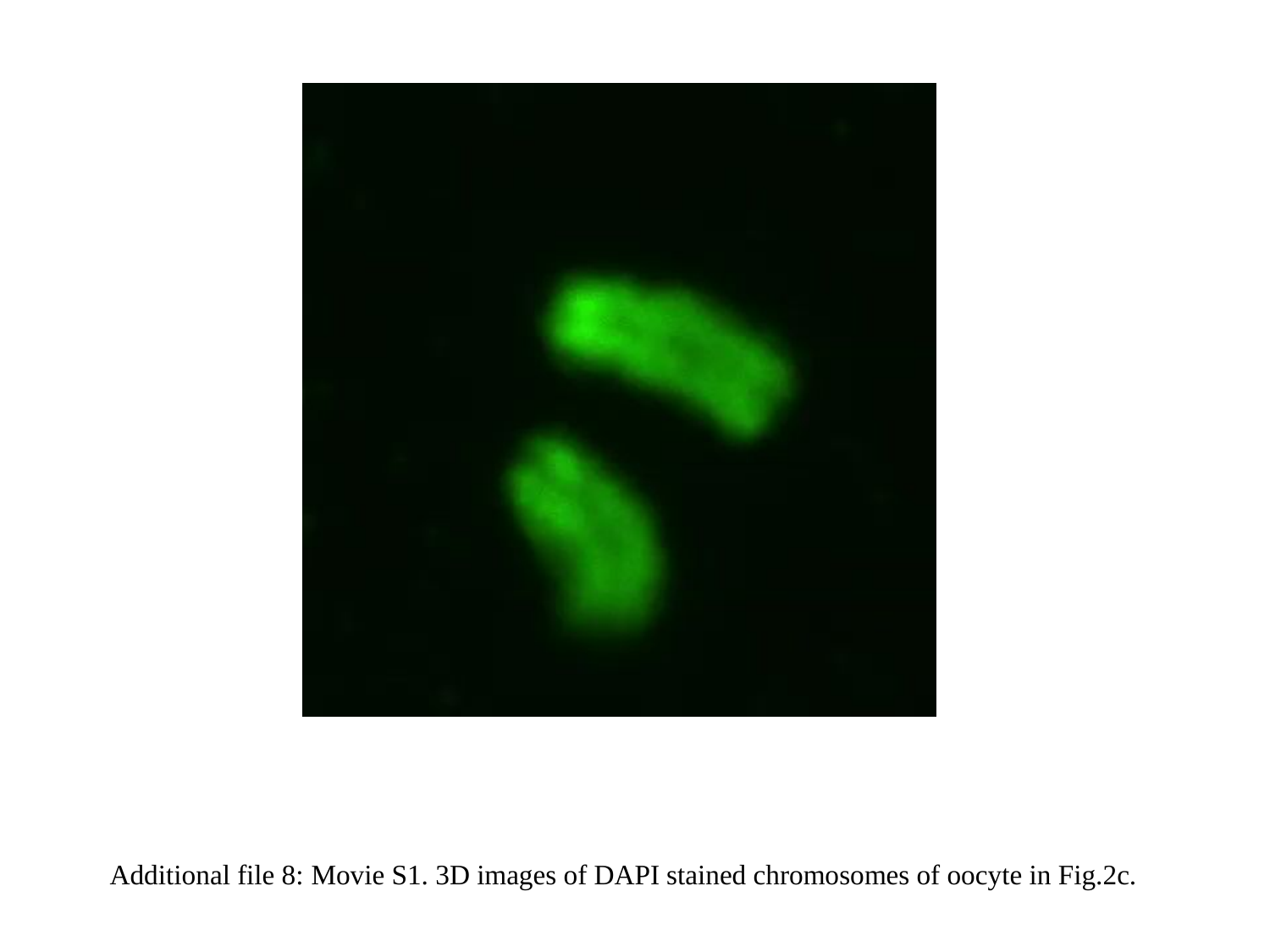

Additional file 8: Movie S1. 3D images of DAPI stained chromosomes of oocyte in Fig.2c.

## Slide 2
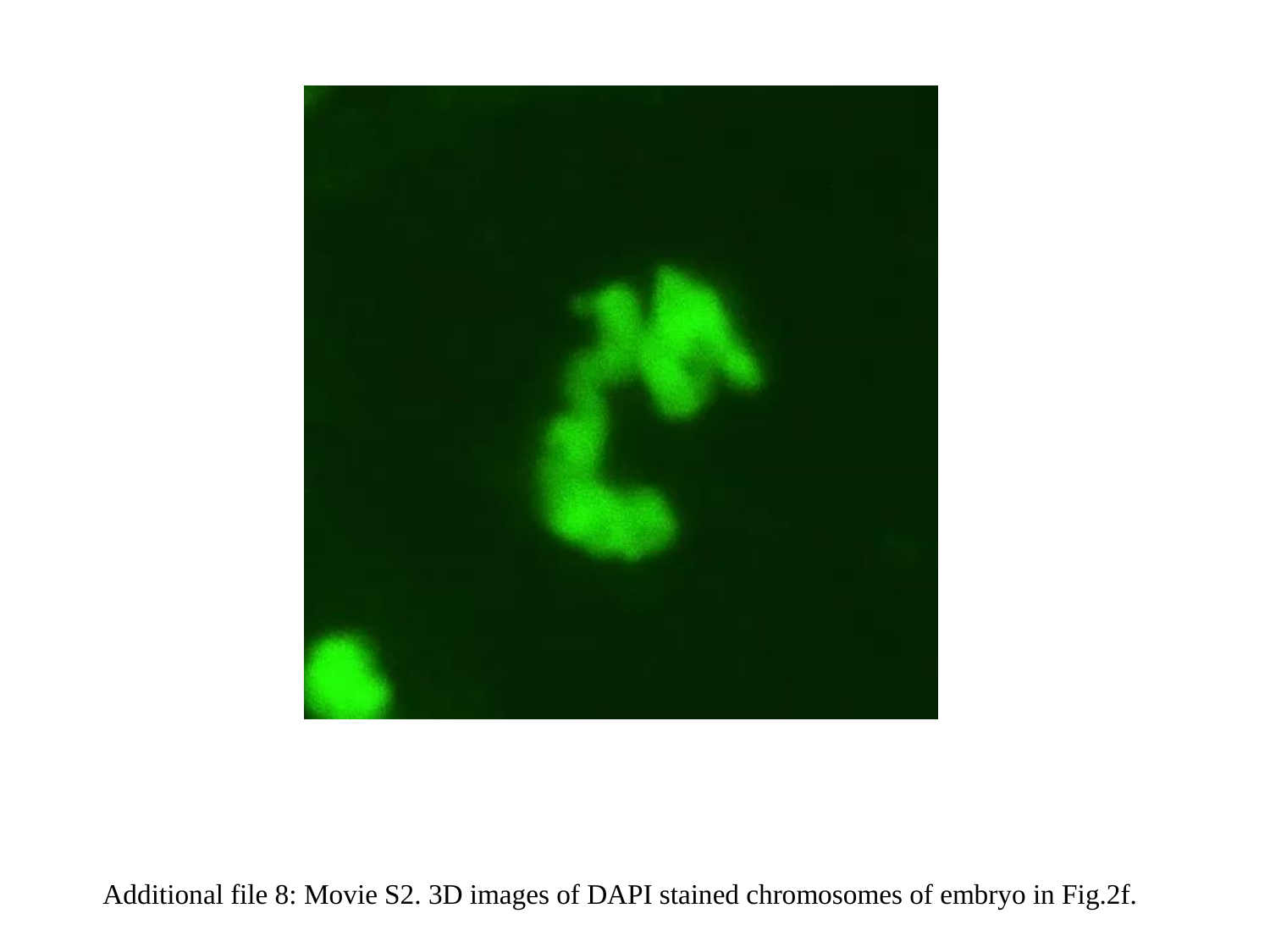

Additional file 8: Movie S2. 3D images of DAPI stained chromosomes of embryo in Fig.2f.
